# An Integrated View of Deubiquitinating Enzymes Involved in Type I Interferon Signaling, Host Defense and Antiviral Activities

**DOI:** 10.3389/fimmu.2021.742542

**Published:** 2021-10-11

**Authors:** Guanghui Qian, Liyan Zhu, Gen Li, Ying Liu, Zimu Zhang, Jian Pan, Haitao Lv

**Affiliations:** ^1^ Institute of Pediatric Research, Children’s Hospital of Soochow University, Suzhou, China; ^2^ Department of Experimental Center, Medical College of Soochow University, Suzhou, China

**Keywords:** deubiquitinating enzymes, type I IFN signaling, ubiquitin, virus infection, innate immunity

## Abstract

Viral infectious diseases pose a great challenge to human health around the world. Type I interferons (IFN-Is) function as the first line of host defense and thus play critical roles during virus infection by mediating the transcriptional induction of hundreds of genes. Nevertheless, overactive cytokine immune responses also cause autoimmune diseases, and thus, tight regulation of the innate immune response is needed to achieve viral clearance without causing excessive immune responses. Emerging studies have recently uncovered that the ubiquitin system, particularly deubiquitinating enzymes (DUBs), plays a critical role in regulating innate immune responses. In this review, we highlight recent advances on the diverse mechanisms of human DUBs implicated in IFN-I signaling. These DUBs function dynamically to calibrate host defenses against various virus infections by targeting hub proteins in the IFN-I signaling transduction pathway. We also present a future perspective on the roles of DUB-substrate interaction networks in innate antiviral activities, discuss the promises and challenges of DUB-based drug development, and identify the open questions that remain to be clarified. Our review provides a comprehensive description of DUBs, particularly their differential mechanisms that have evolved in the host to regulate IFN-I-signaling-mediated antiviral responses.

## Introduction

Pathogen invasions are responsible for many diseases and exert extensive effects on human health ranging from mild to potentially fatal infections. Critically, the prevalence of certain viruses, such as SARS-CoV-2, can even pose a serious threat to global human health (1). The host’s immune system evolved as the first line of defense against the invasion of microbial pathogens and can also trigger various immune responses through dynamic interactions with differential cellular components (2, 3). Among all the signaling pathways examined, much attention has been given to the signaling events triggered by one class of molecules during the activation of innate immune responses, pattern-recognition receptors (PRRs). Innate immune responses are rapidly initiated when host cellular PRRs, such as Toll-like receptors (TLRs), RIG-I-like receptors (RLRs), NOD-like receptors (NLRs), and DNA sensors encounter pathogen-associated molecular patterns (PAMPs) of fungal, bacterial, or viral origin (4, 5). Toll-like receptors, including TLR3, TLR4, TLR7, TLR8, and TLR9 can sense endosomal nucleic acids derived from pathogens and infected apoptotic cells. Specifically, TLR3 and TLR7/8 recognize double-stranded RNA (dsRNA) and single-stranded RNA (ssRNA), respectively, whereas TLR9 detects unmethylated CpG double-stranded DNA species (6). The activation of TLR3, TLR4, TLR7, TLR8, and TLR9 leads to activation of the adapter myeloid differentiation 88 (MyD88)-dependent pathway, which causes IRF7 activation through a TRAF6-dependent mechanism (TLR7/8/9) or the Toll/interleukin-1 (IL-1) receptor-domain-containing adapter-inducing IFNβ (TRIF)-dependent pathway and thus leads to IRF3 and IRF7 activation through a TBK1-dependent mechanism (TLR3/4) (7–9). RLRs are another critical sensor of virus infection. These protein family members include retinoic acid-inducible gene I (RIG-I, also known as Ddx58), melanoma differentiation-associated protein 5 (MDA5, also known as Ifih1 or Helicard), and laboratory of genetics and physiology protein 2 (LGP2) (10). Viral 5’ ppp RNA, and longer double-stranded (ds) RNA are often recognized by RIG-I and MDA5, respectively, and both proteins share two N-terminal caspase activation and recruitment domains (CARDs), which are needed for interaction with the mitochondrial antiviral signaling protein (MAVS, also termed IPS-1, VISA or CARDIF). The interacting components then activate MAVS and TNF receptor-associated factors (TRAF)-mediated downstream signaling during virus infection (11). Ultimately, the viruses recognized by different host sensors induce antiviral responses by regulating multiple signaling pathways, which are characterized by rapid gene expression of inflammation-inducing molecules and/or cytokines, including interferons (12–15).

**Figure 1:**
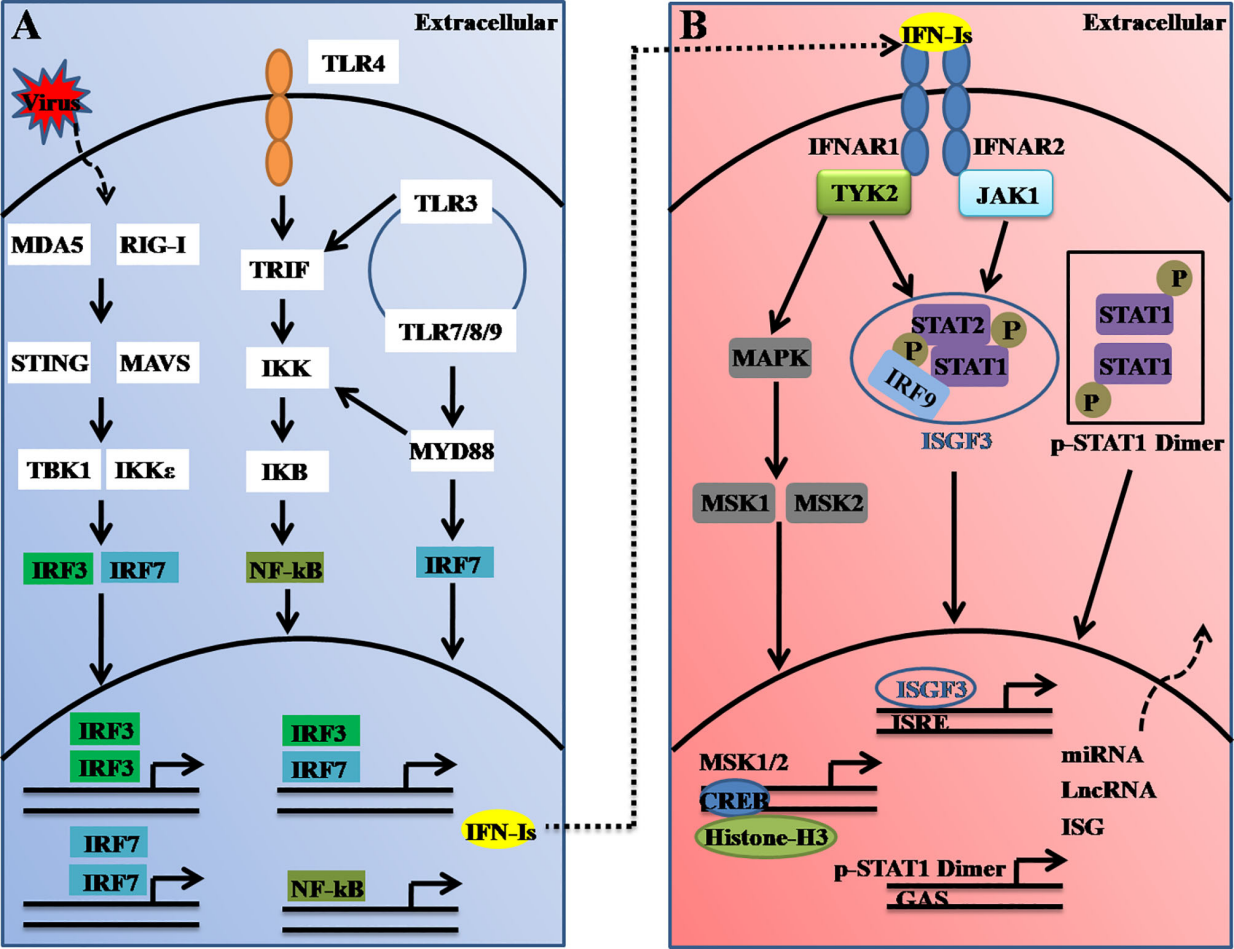
Schematic illustration of type I interferon (IFN-I) induction and receptor signaling pathways. **(A)** Type-I IFNs are induced upon virus nucleic acid recognition by a variety of PRRs, including TLRs and cytosolic nucleic acid sensors. The activation of PRRs causes the nuclear translocation of IRFs or NF-κB, which bind to the promoter region of IFN-Is and thus induce their transcription. IRF3- and IRF7-mediated IFN-I production could be regulated by STING (*via* cGAS), RIG-I, MDA5, TLR3, and TLR4 (through TRIF), whereas the ligand engagement of TLR7/8 and TLR9 activates IRF7 *via* MyD88. **(B)** Secreted interferons bind to the IFNAR complex composed of IFNAR1 and IFNAR2, which causes cross-phosphorylation of JAK1 and TYK2 and further activation of STAT homo/heterodimers to control distinct expression profiles. ISGF3, which comprise of STAT1, STAT2, and IRF9, binds to the IFN-stimulated response element (ISRE). TYK2 activates MAPK and MSK1/2. Nuclear MSK1/2 further phosphorylates CREB and induces the transcriptional induction of hundreds of genes or noncoding RNAs.

Type I IFNs (also called IFNα/β or IFN-Is), which serve as the first line of host defense against virus infection can be induced in almost all cells in the body. A dysregulated interferon-response is thus associated with many diseases, such as autoimmune diseases (16), infectious diseases (17), and the recent severe coronavirus diseases, which have caused a major ongoing pandemic worldwide (18). The critical cytosolic DNA sensor, cyclic guanosine monophosphate-adenosine monophosphate (cGAMP) synthase (cGAS) often recognizes viral DNA and triggers downstream immune responses through the molecule stimulator of interferon genes (STING, also known as MITA, MPYS, ERIS, or TMEM173) (19). STING further activates TRAFs, which in turn activate TANK-binding kinase 1 (TBK1) or IκB kinase (IKK), and this activation leads to the activation of nuclear factor-kappa enhancer-binding protein (NF-κB) or interferon regulatory factor 3 or 7 (IRF3 or IRF7, respectively). The activated IRF3 and IRF7 complex ultimately translocates the nucleus, which leads to the transcriptional induction of multiple IFNs (**Figure 1A**) (20).

Furthermore, the secreted IFN-Is bind to and signal through a heterodimeric transmembrane receptor composed of the subunits IFNAR1 and IFNAR2. The ligation of IFNAR activates the receptor-associated protein tyrosine kinases Janus kinase 1 (JAK1) and tyrosine kinase 2 (TYK2). In the canonical IFNAR-mediated downstream signaling pathway, activated JAK1 and TYK2 induce phosphorylation of the signal transducer and activator of transcription 1 (STAT1) and STAT2 molecules present in the cytosol, which leads to the dimerization, nuclear translocation, and binding of these molecules to IRF9 to form the ISG factor 3 (ISGF3) complex. This complex then enters the nucleus and binds to DNA sequences termed interferon-sensitive response elements (ISREs) (with the consensus sequence TTTCNNTTTC), which results in induction of the transcription of several hundred IFN-stimulated genes (ISGs), including Mx1, OAS, STAT1, interferon-regulatory factors (IRFs) and other antiviral genes (21) (**Figure 1B**). These ISGs function to induce an antiviral state within the cell. Thus, it can be concluded that host antiviral efficiencies are tightly regulated not only at the virus-induced IFN-I production level but also at the interferon receptor-mediated downstream signaling level.

Currently, post-translational modifications (PTMs), which involve the covalent linkage of new functional groups to amino acid chains, have remarkably expanded the functions of proteins. Over the years, an increasing number of studies have uncovered that PTMs also play pivotal roles during host innate immune responses upon virus infection (22, 23). In particular, ubiquitination (also known as ubiquitylation or ubiquitinylation) events in which 8.5-kDa ubiquitin (Ub) is conjugated to one or more lysine residues of proteins are broadly involved in antiviral signaling by regulating the stability, folding, and location of proteins or by interacting with other proteins in the signaling transduction pathway (22, 24). In general, ubiquitination involves three sequential steps: an initial activation step catalyzed by the Ub-activating enzyme (E1), an intermediate step in which Ub is covalently linked to a conjugating enzyme (E2), and a final specific step in which Ub reaches its ultimate destination of the substrate amino group through a reaction catalyzed by a ligase enzyme (E3) (25–27). Substrate-conjugated ubiquitin can be modified by additional Ub molecules to build polyubiquitin chains. The C-terminal carboxyl group of the distal Ub moiety is covalently attached to either the first methionine (M1) of the proximal Ub moiety or one of the seven lysine (K) residues K6, K11, K27, K29, K33, K48, and K63 to result in the formation of linear Ub chains or polyubiquitin chains (28–30). Homotypic polyubiquitin chains are often referred to as a single type of polyubiquitin linkage, whereas heterotypic polyubiquitin chains are characterized by the presence of at least two different types of linkages within the same polymer (31).

**Figure 2:**
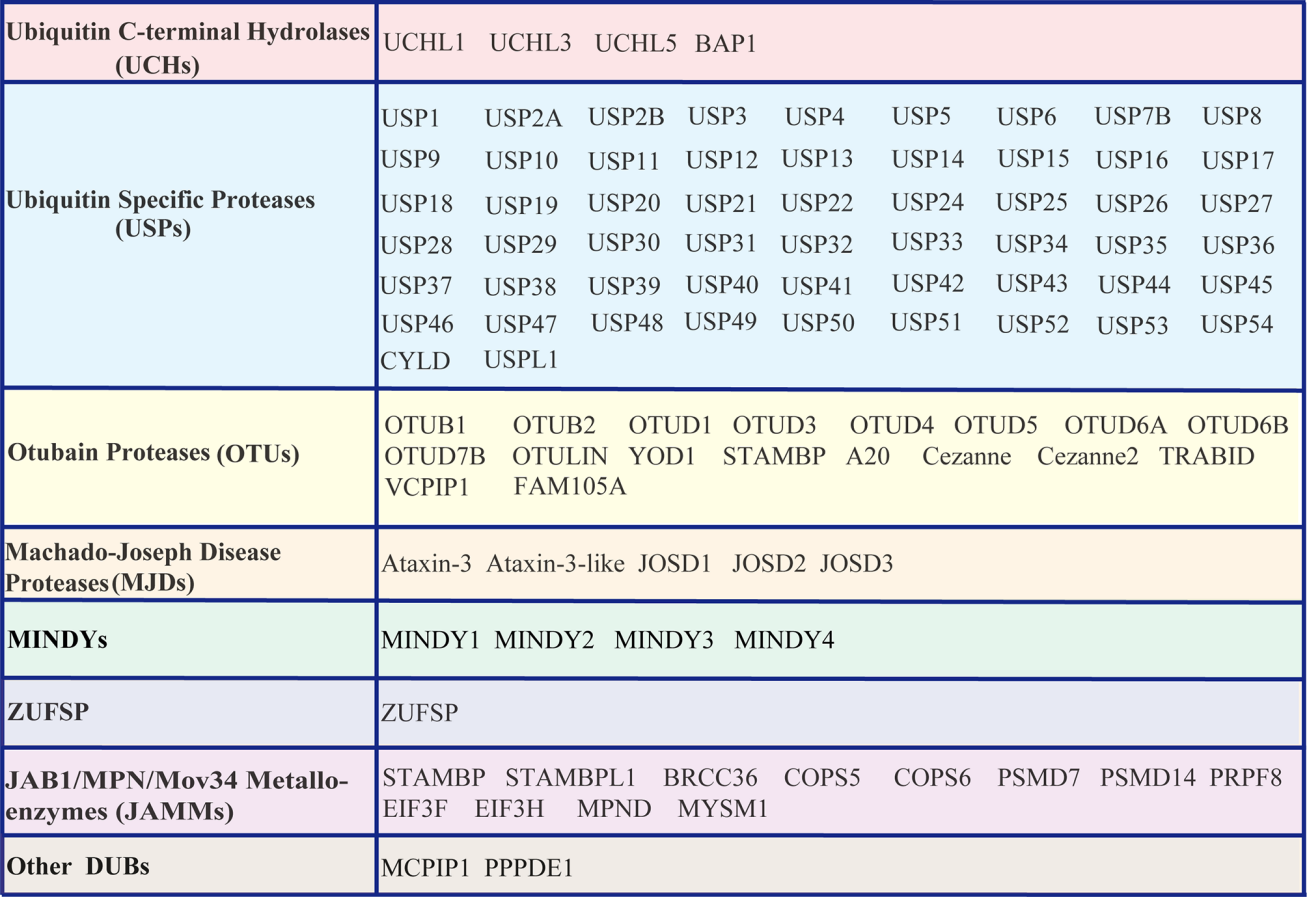
List of DUBs identified in the human genome. These DUBs are categorized into at least seven subfamilies, namely, Ub carboxyl-terminal hydrolases (UCHs), Ub-specific proteases (USPs), ovarian tumor proteases (OTUs), Machado-Joseph disease proteases (MJDs), motifs interacting with Ub (MIU)-containing novel DUB family members (MINDYs), zinc fingers with UFM1-specific peptidase domain protein/C6orf113/ZUP1 (ZUFSP), JAB1/MPN/MOV34 metalloenzyme family members (JAMMs, also termed MPN+), and other newly identified members.

Similar to other PTMs, ubiquitination is reversible, and the reversal process is implemented by an array of proteases termed deubiquitinating enzymes (DUBs) or deubiquitinating peptidases. Approximately 100 DUBs encoded in the human genome. These DUBs have been categorized into at least seven families based on their homology domains and cleavage preferences: namely, ubiquitin-specific proteases (USPs), ubiquitin C-terminal hydrolases (UCHs), ovarian tumour proteases (OTUs), Machado-Joseph disease protease family members (MJDs), the motif interacting with the Ub (MIU)-containing novel DUB family (MINDYs), the JAB1/MPN/MOV34 metalloenzyme family (JAMMs, also termed MPN+), zinc fingers with UFM1-specific peptidase domain proteins (ZUFSPs), and other members identified recently (32–36) (**Figure 2**). These DUBs often contain a catalytic domain surrounded by one or more accessory domains, and some of these domains contribute to Ub binding and target recognition (37). One of the best-characterized functions of DUBs is the removal of monoubiquitin and polyubiquitin chains from proteins, thus ensuring that the Ub-proteasome system (UPS) functions properly and recycles free Ub for reuse to maintain the homeostasis of the polyubiquitin pool (38, 39). Analogous to the dynamic and crucial roles of ubiquitination events shown previously, DUB-mediated deubiquitination events also play important roles in the antiviral innate immune response (23, 24, 40, 41). Here, we summarize the differential regulatory roles of human DUBs involved in the IFN-I signaling transduction pathway during viral infections. Integrated analyses of DUBs involved in the IFN-I signaling transduction pathway might improve our understanding of their diverse regulatory mechanisms and host antiviral activities, and facilitate the development of therapeutic targets to improve host antiviral efficiency in the future.

## DUBs Regulate Virus-Induced IFN-I Production and Antiviral Activities

**Figure 3:**
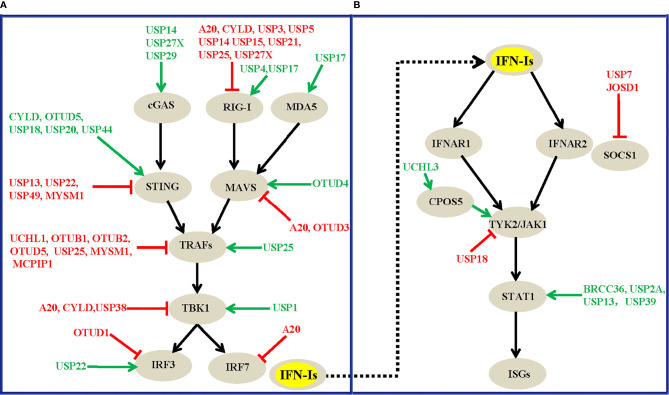
Overview of DUBs that modulate the virus-induced IFN-I production signaling **(A)** and the IFNAR-mediated downstream signaling transduction pathway **(B)**. The green arrows and red lines respectively indicate the positive and negative regulatory roles of each DUB involved.

Constitutively expressed RLRs often reside in the cytoplasm of uninfected cells in an auto-repressed, inactive state (42). However, upon viral infection, the master regulators RIG-I and MDA5 are rapidly activated and then induce the transcriptional induction of multiple IFNs. Additionally, mice lacking RIG-I or MDA5 are highly susceptible to infection and fail to produce IFN-I and proinflammatory cytokines (43, 44). Given the importance of RIG-I and MDA5 in the RLR signaling pathways, the functions of the two proteins are affected by multiple PTM events, such as phosphorylation and ubiquitination. For instance, several E3 ligases, such as TRIM25 (45), RNF135 (46), RNF125 (47), RNF122 (48), TRIM40 (49), CHIP (50), and c-Cbl (51), regulate RIG-I signaling by modulating Ub chains from various signaling proteins. Among these, the K63-linked ubiquitination of RIG-I often represents a critical step in promoting the activation of IFN-I signaling (45, 46, 52). Intriguingly, IFN stimulation could also promote an increase in the expression level of RIG-I. Thus, the protein turnover and activity of RIG-I must be tightly regulated to ensure restoration to homeostasis and to avoid hyperactivation of IFN and cytokine signaling. To the best of our knowledge, at least nine DUBs, A20, CYLD, USP3, USP5, USP14, USP15, USP21, USP25, and USP27X, have been proposed to counteract the K63-linked ubiquitination of RIG-I and, thereby attenuate downstream signaling and IFN-β production (**Table 1** and **Figure 3**) (58, 76, 93). However, unlike the nine above-mentioned Dubs, USP4 and USP17 are the two DUBs that positively regulate virus-induced IFN-I signaling by increasing the stability of RIG-I (**Table 1** and **Figure 3**). Congruently, the overexpression of USP4 or USP17 significantly promotes virus-induced IFN production and thereby restricts virus replication, whereas the knockdown of USP4 or USP17 has the opposite effect (77, 87). Moreover, DUBs also exhibit different functions under different contexts. For example, the deubiquitinating enzyme USP15 negatively regulates virus-induced IFN-I production by targeting RIG-I (84). However, USP15 has also been identified to positively regulate type I IFN responses by decreasing the polyubiquitination level of TRIM25 (85, 86). Because the function of DUBs can be altered by various PTMs under differential contexts (123), the discrepancy that USP15 exerts both positive and negative effects may arise from the context-specific PTM of USP15 itself, which may allow dynamic fine-tuning of the signaling. Among the DUBs that interact with STING, five members, namely CYLD, OTUD5, USP18 (also termed UBP43), USP20, and USP44, have been demonstrated to promote IFN-I production and antiviral responses. In addition, although USP18 cannot deubiquitinate STING itself, it can recruit USP20 to deubiquitinate STING and thereby suppresses virus-induced IFN-I production (91). However, the other four DUBs (USP13, USP22, USP49, and MYSM1) inhibit IFN-I-mediated antiviral activity by deubiquitinating K27- or K63-linked polyubiquitin chains of STING (40, 81, 103, 105). Consistent with this observation, USP13- and USP49-deficient mice are more resistant to lethal herpes simplex virus type 1 (HSV-1) infection than their wild-type (WT) littermates (81, 103). In addition, MYSM1 interacts with STING to cleave STING ubiquitination and attenuate the pathway, and MYSM1-deficient mice exhibit tissue damage and high mortality upon virus infection (105). Moreover, MAVS activation and aggregation, which is promoted by K63-linked ubiquitination catalyzed by TRIM31 (124), are counteracted by OTUD3 (65). In addition, OTUD3-deficient mice also exhibit decreased morbidity after infection with vesicular stomatitis virus (VSV), which might result from increased production of cytokines and decreased viral replication (65). In addition, both OTUD3 and A20 negatively regulate the IFN-mediated antiviral response by modulating the polyubiquitination level of MAVS (125, 126). However, OTUD4 positively regulates IFN signaling and enhances host antiviral activities by deubiquitinating K48-Ub on MAVS (66).

**Table 1:** Summary on human DUBs involved in the regulation on IFN-I signaling and antiviral responses.

DUB	Substrate	Ub Model	Effect	Specific Event	References
A20	RIG-I	NA	–	Suppressing VSV through inhibition on RIG-I	(53)
A20	MAVS	NA	–	Suppressing VSV through inhibition on MAVS	(53)
A20	IRF7	K63	–	Deubiquitinating K63-Ub on IRF7 in 293 cell	(54)
A20	TRAF6	K63	NA	Deubiquitinating K63-Ub on TRAF6 in HEK293T cells	(55)
A20	IKK-γ	NA	NA	Interacting with ubiquitinated NEMO, inhibiting IKK phosphorylation and NF-κB activation	(56)
CYLD	IKK-γ	M1	NA	Suppressing NF-κB signaling	(57)
CYLD	RIG-I	K63	–	Deubiquitinating K63-Ub on RIG-I to decrease IFN production	(58)
CYLD	MAVS	NA	–	Interacting with but not deubiquitinating MAVS to negatively regulate IFN production	(58)
CYLD	TBK1	K63	–	Deubiquitinating K63-Ub on TBK1, negatively regulating RIG-I-mediated antiviral response	(58)
CYLD	STING	K48	+	Deubiquitinating K48-Ub on STING, promoting the innate antiviral response	(59)
UCHL1	TRAF3	K63	–	Deubiquitinating K63-Ub on TRAF3 in HEK293T cell, negatively regulating virus-induced IFNs production	(60)
OTUB1	TRAF3	Ub	–	Deubiquitinating Ub on TRAF3, negative regulating virus-induced IFNs signaling	(61)
OTUB2	TRAF6	Ub	–	Deubiquitinating Ub on TRAF6, negatively regulating virus-induced IFNs signaling	(61)
OTUD1	IRF3	K63	NA	Deubiquitinating K63-Ub on IRF3, inhibiting IRF3 nuclear translocation and transcriptional activity	(62)
OTUD1	IRF3	K6	–	Deubiquitinating the viral infection-induced K6-linked ubiquitination on IRF3	(63)
OTUD1	SMURF1	K48	–	Deubiquitinating K48-Ub on SMURF1, causing degradation on MAVS/TRAF3/TRAF6	(64)
OTUD3	MAVS	K63	–	Deubiquitinating K63-Ub on MAVS, inhibiting innate antiviral immune responses	(65)
OTUD4	MAVS	K48	+	Deubiquitinating K48-Ub on MAVS, promoting antiviral responses	(66)
OTUD4	MyD88	K63	NA	Suppressing TLR/NF-κB signaling	(67)
OTUD5	TRAF3	K63	–	Deubiquitinating K63-Ub on TRAF3, suppressing type I IFN production in HEK293 cells	(68)
OTUD5	STING	K48	+	Deubiquitinating K48-Ub on STING, promoting innate antiviral immunity.	(69)
OTUD7B	RIPK1	K48&K63	NA	Deubiquitinating K48 and K63-Ub on RIPK1	(70, 71)
OTUD7B	TRAF3	K48	NA	Deubiquitinating K48-Ub on TRAF3, inhibiting TRAF3 proteolysis, preventing NF-κB activation	(72)
OTUD7B	TRAF6	K63	NA	Deubiquitinating TRAF6 in HUVECs	(73)
USP1	TBK1	K48	+	Inhibiting TBK1 degradation, promoting RIG-I- induced IRF3 activation and IFN-β secretion	(74)
USP2B	TBK1	K63	–	Deubiquitinating K63-Ub on TBK1 to inhibit TBK1 kinase activity	(75)
USP3	RIG-I	K63	–	Deubiquitinating K63-Ub on RIG-I, to convert RIG-I to its inactive form in 293T	(76)
USP4	RIG-I	K48	+	Deubiquitinating K48-Ub on RIG-I to stabilize RIG-I	(77)
USP4	TRAF6	K48	NA	Deubiquitinating K48-Ub on TRAF6, positively regulating RLR-induced NF-κB activation	(78)
USP5	RIG-I	K48	–	Increasing the K48-Ub on RIG-I after SeV infection	(40)
USP7	TRIM27	K48	–	USP7 knockout destabilizes TRIM27, which increase TBK1 turnover and IFNs signaling	(79)
USP7	NF-κB	K48	NA	Stabilizing NF-κB, increasing NF-κB transcription	(80)
USP13	STING	K27	–	Inhibiting the recruitment on TBK1 to STING by deubiquitinating K27-Ub on STING	(81)
USP14	RIG-I	K63	–	Deubiquitinating K63-Ub on RIG-I in 293T cell	(82)
USP14	cGAS	K48	+	Recruited by TRIM14 to stabilize cGAS, functions as a positive feedback loop on cGAS signaling	(83)
USP15	RIG-I	K63	–	Deubiquitinating K63-Ub on RIG-I in HEK-293T cells	(84)
USP15	TRIM25	K48	+	Deubiquitinating K48-Ub on TRIM25 to maintain TRIM25 in an inactivate state	(85)
USP15	TRIM25	Ub	+	Deubiquitinating Ub on TRIM25 in haematopoietic cells and resident brain cells	(86)
USP17	RIG-I	K48&K63	+	Deubiquitinating K48-Ub on RIG-I	(87)
USP17	MDA5	K48&K63	+	Deubiquitinating K48-Ub and K63-Ub on MDA5	(87)
USP18	ISG15	NA	–	Recruiting USP20 to form a complex with STING independently on DUB activity	(88)
USP18	TAK1	K63	NA	Suppressing TLR/NF-κB signaling	(89)
USP19	TRIF	K27	–	Deubiquitinating K27-Ub on TRIF to impair the recruitment of TRIF to TLR3/4	(90)
USP20	STING	K48	+	Deubiquitinating K33- or K48 Ub on STING together with USP18	(91, 92)
USP21	RIG-I	K63	–	Deubiquitinating K63-Ub on RIG-I in HEK 293T cells	(93)
USP22	STING	K27	–	Deubiquitinating K27-Ub on STING by recruiting USP13	(40)
USP22	IRF3	K48	+	Stabilizing KPNA2, promoting IRF3 nuclear translocation	(94)
USP25	RIG-I	K48&K63	–	Deubiquitinating RIG-I in HEK-293T cells	(95)
USP25	TRAF3	K48&K63	–	Deubiquitinating TRAF3 in HEK-293T cells	(95)
USP25	TRAF6	K48&K63	–	DeubiquitinatingTRAF6 in HEK-293T cells	(95)
USP25	TRAF3	K48	+	Deubiquitinating K48-Ub in BMDCs and MEFs	(96)
USP25	TRAF6	K63	+	Deubiquitinating Ub on TRAF6	(96)
USP27X	RIG-I	K63	–	Deubiquitinating K63-Ub on RIG-I	(97)
USP27X	cGAS	K48	+	Deubiquitinating K48-Ub on cGAS to stabilize cGAS	(98)
USP29	cGAS	K48	+	Deubiquitinating and stabilizing cGAS to promote innate antiviral responses against DNA viruses	(99)
USP31	TRAF2	K48	NA	Deubiquitinating K48-Ub and stabilizing TRAF2	(100)
USP38	TBK1	K33	–	USP38 knockout increases K33-linked Ub but abrogates the K48-mediated degradation on TBK1	(101)
USP44	STING	K48	+	Preventing STING from proteasome-mediated degradation	(102)
USP49	STING	K63	–	Deubiquitinating K63-Ub on STING, inhibiting STING aggregation and the recruitment on TBK1	(103)
MYSM1	TRAF3	K63	–	Deubiquitinating K63-Ub on TRAF3	(104)
MYSM1	TRAF6	K63	–	Deubiquitinating K63-Ub on TRAF6	(104)
MYSM1	STING	K63	–	Deubiquitinating K63-Ub on STING	(105)
MCPIP1	TRAFs	K48&K63	–	Deubiquitinating TRAFs and inhibiting IRF3 nuclear translocation in HEK293T and HeLa cells	(106, 107)
ATXN3	HDAC3	K48&K63	+	Deubiquitinating K48- and K63-Ub on HDAC3 in 293T cells	(108)
BRCC36	IFNAR1	K63	+	Deubiquitinating K63-Ub on IFNAR1 to sustain the turnover of IFNAR1 in 2fTGH cells	(109)
BRCC36	STAT1	K63	+	Maintaining the STAT1 levels by recruiting USP13 to antagonize the SMURF1-mediated degradation on STAT1	(110)
USP2A	p-STAT1	K48	+	Inhibiting K48-Ub-linked ubiquitination and degradation on pY701-STAT1 in the nucleus	(111)
USP5	SMURF1	K63	–	Deubiquitinating K63-Ub on SMURF1, inhibiting the IFN-mediated antiviral activity	(112)
USP7	SOCS1	Ub	–	Enhancing SOCS1 protein stability *via* deubiquitination effects	(113)
USP12	CBP	NA	+	Regulating CBP and TCPTP independently on the deubiquitinase activity	(114)
USP13	STAT1	K48	+	Deubiquitinating and stabilizing STAT1	(115)
USP18	JAK1	NA	–	Interacting with IFNAR2, restricting its interaction with JAK, inhibiting the tyrosine kinase activity of JAK	(116, 117)
USP39	STAT1	K6	+	Decreasing K6-linked Ub on STAT1 for degradation	(118)
MCPIP1	NA	Ub	+	Promoting IFN signaling by increasing ISRE promoter activity and ISG expression	(119)
JOSD1	SOCS1	K48	–	Deubiquitinating K48-Ub on SOCS1	(120)
COPS5	TYK2	NA	+	Stabilizing IFNAR by antagonizing the NEDD8 pathway	(121)
UCHL3	COPS5	K48&K63	+	Deubiquitinating K48- and K63-linked Ub on COPS5, increasing the IFNAR1 turnover in 293T cells	(122)

NA, not available; Ub model, the deubiquitination type on each DUB acting on the targeted proteins; effects, the DUBs positively (＋) or negatively (－) regulate type I IFN signaling-mediated antiviral activity.

Among the DUBs that interact with cGAS or MDA5, USP27X (98) and USP29 (99) stabilize cGAS and thus positively regulate IFN production and antiviral activities. The knockout of USP27X in mouse macrophages significantly impairs innate antiviral responses (98), whereas the knockdown or knockout of USP29 severely impairs HSV1- or cytosolic DNA-induced expression of IFN-Is and proinflammatory cytokines (99). In addition, USP17 promotes virus-induced IFN-I production by decreasing the polyubiquitination level of MDA5 (87). Notably, UCHL1, OTUB1, OTUB2, OTUD5, USP25, MYSM1, and MCPIP1 (**Figure 3A**) negatively regulate virus-induced IFN-I production and antiviral activities by cleaving K63-linked or other types of polyubiquitin chains from TRAF3 or TRAF6. Regarding the kinase TBK1, a previous study showed that the T cell anergy–related E3 Ub ligase RNF128 catalyzes the K63-linked polyubiquitination of TBK1, which causes TBK1 and IRF3 activation, and IFN-β production (127). The E3 ligases DTX4, Triad3A, and TRIP have also been identified to conjugate K48-linked polyubiquitin chains on TBK1, which results in TBK1 degradation and subsequent inhibition of IFN-Is (128–130). However, DUBs cleave the polyubiquitin chains of TBK1 to reverse the ubiquitination process mediated by E3 ligases (**Table 1** and **Figure 3**). For example, CYLD removes polyubiquitin chains from TBK1 and RIG-I and thus inhibits the IRF3 signaling pathway and IFN production triggered by RIG-I; conversely, CYLD knockdown enhances this response (58). Similarly, USP38 negatively regulates IFN-I signaling by targeting the active form of TBK1 for degradation *in vitro* and *in vivo* (101). USP19 suppresses virus-induced IFN-I production by targeting TIR domain-containing adaptor inducing interferon-β (TRIF, also known as Ticam1), which is an adaptor required for innate immune responses mediated by TLR3 and TLR4 (90). Together, these results indicate that the crosstalk between IFN-I and the Toll-like signaling pathway functions intricately in regulating host antiviral activities.

Altogether, the diversity of the mechanisms of DUB regulation enables the tight regulation of their function, which ensures an appropriate innate immune response against virus infections. To the best of our knowledge, at least twenty-four DUBs, A20, UCHL1, OTUB1, OTUB2, OTUD1, OTUD3, OTUD5, USP2B, USP3, USP5, USP7, USP13, USP14, USP15, USP18, USP19, USP21, USP22, USP25, USP27X, USP38, USP49, MYSM1, and MCPIP1, have so far been identified to negatively regulate virus-induced IFN-I production and antiviral activity. In contrast, DUBs, including CYLD, OTUD4, OTUD5, USP4, USP15, USP17, USP20, USP27X, USP29, and USP44, have been suggested to positively regulate host antiviral responses by targeting various substrates in this pathway (**Table 1**). These DUBs mainly regulate the polyubiquitination levels of RIG-I, STING, MAVS, TRAFs, and TBK1, which function at different levels of this pathway (**Figure 3A**), and this finding implies the physiological importance of these master proteins in innate immunity during viral infections. Of note, one DUB might target different proteins in the same pathway, whereas the same substrate might also be regulated by more than one DUB, which suggest the existence of dynamic and complex crosstalk between DUBs and substrates involved in IFN-I signaling-mediated antiviral activities. However, why so many DUBs are involved in host immune responses during viral infections remains unclear. One possible reason is that different DUBs may exert differential functions in response to various stimuli, and some of the Dubs might function redundantly in specific contexts. Second, the experimental tools and research biases might also contribute to the diverse roles of DUBs that have been identified. Moreover, some findings are only based on cell lines and overexpression systems and need to be confirmed *in vivo* and with genetic models in the future.

## DUBs in IFNAR-Mediated Downstream Signaling and the Antiviral Response

In addition to their roles in virus-induced IFN-I production signaling, signaling molecules downstream of the IFN receptor play pivotal roles in affecting host antiviral efficiency. Because increasing the dosage of IFNs alone cannot significantly improve host antiviral efficiency, it has been proven that IFNs can induce ubiquitin-dependent degradation of the IFNAR receptor, which leads to a restriction effect on host antiviral activities (131, 132). Consequently, it is similarly important to investigate the roles of DUBs involved in the IFNAR-mediated downstream signaling pathway. However, compared with the relatively large number of DUBs that regulate virus-induced IFN-I production (**Figure 3A**), the number of DUBs that have been implicated in IFNAR-mediated downstream signaling has rarely been explored (**Figure 3B**). In most cases, the regulatory effects of DUBs are mainly focused on the STAT1 protein, which functions as an essential transcription factor in IFNAR1-mediated downstream signaling. The ubiquitination and deubiquitination regulation events of STAT1 and its associated effects on the innate immune response have been increasingly investigated in recent years. For example, the three deubiquitinating enzymes BRCC36, USP13, and USP39 interact with STAT1 and decrease the K63-, K48- and K6-linked polyubiquitin chains of STAT1 respectively (110, 115, 118). These three DUBs positively regulate IFN-mediated antiviral activities and have been proposed to antagonize the degradation rate of STAT1 mediated by two E3 ligases, SLIM (133) and SMURF1 (134). More specifically, BRCC36 deficiency leads to a rapid downregulation of STAT1 during viral infection, whereas complementation by BRCC36 can rescue the STAT1 expression levels and suppress virus infection (110). BRCC36 sustains STAT1 protein turnover by recruiting USP13 to form a balanced complex to antagonize the SMURF1-mediated degradation of STAT1 (110). More specifically, USP13 positively regulates the antiviral activity of IFNα against DEN-2 virus replication by deubiquitinating and stabilizing STAT1 (115). Intriguingly, although USP39 was previously shown to not have deubiquitinase activity, recent studies have shown that USP39 combines with STAT1 and stabilizes its expression level by preventing the K6-linked polyubiquitination of STAT1 which promotes its degradation, and USP39 thus positively regulates IFN-I-mediated antiviral activities (118). Notably, IFN treatment could also promote USP2A to interact with pY701-STAT1 and maintain the pY701-STAT1 levels in the nucleus, which enhances IFN signaling-mediated antiviral activity (111). Unlike USP2A, the deubiquitinating enzymes BRCC36, USP13, and USP39 positively regulate IFN activities by attenuating the polyubiquitination level of STAT1, and this process is independent of IFN treatment, which suggests divergent functional roles of these DUBs under differential contexts.

Additionally, ATXN3 does not affect IFN-I production during viral infection but positively regulates IFNAR1-mediated downstream signaling by targeting HDAC3 (108). Another DUB, UCHL3, also positively regulates IFN-I-mediated antiviral activity by increasing the stability of COPS5 and IFNAR1 (121, 122). Moreover, both USP7 and JOSD1 have been identified as negative regulators of IFNAR1-mediated downstream signaling by decreasing the polyubiquitin expression level of SOCS1 and thereby enhancing the turnover of SOCS1, which is a potent suppressor of IFN-I signaling (135). The IFN-inducible deubiquitinase USP18, which functions as one of the most important DUBs in IFN signaling, can downregulate type I IFN signaling by blocking the interaction between JAK1 and IFNAR2 (88, 89, 117). In addition, USP18 has enzymatic activity in cleaving the covalently conjugated 15-kDa protein encoded by interferon-stimulated gene 15 (*ISG15*) from its targeted substrates (136), and USP18 gene-knockout mice exhibit increased susceptibility to *Salmonella typhimurium* or *Mycobacterium tuberculosis* pathogen infections (137). Intriguingly, USP18 also acts as a negative regulator of microglia activation in mice. USP18 deficiency in microglial causes destructive interferonopathy in the mouse brain, suggesting that USP18 plays a protective role in microglia function by regulating the IFNAR pathway (138). Therefore, multiple DUBs are involved in regulating IFNAR-mediated downstream signaling during viral infection. However, whether other DUBs are similarly involved remains unknown, and the connection of DUBs with immune disorders and other related diseases still needs further research.

## DUBs Regulate IFN-I-Mediated Antiviral Responses *via* Their Protease Activity

Because DUBs are proteases, it is often speculated that the DUBs functioning in antiviral immunity are dependent on their deubiquitinating enzyme activities. The Ub chains of each substrate involved in IFN-I signaling are cleaved by various DUBs through either endo- or exo cleavage activity. Although the determination of whether a DUB cleaves with endo- or exo-cleavage activity seems difficult, several studies have shown that this activity relies on both the DUB structure and the type of Ub linkage (39). Indeed, the presence of seven internal lysine residues of the Ub (K6, K11, K27, K29, K33, K48, and K63) and the α-amino-terminus of methionine1 (Met1) enable the modification of target proteins with different types of polyubiquitin chains (conjugation of Ub molecules *via* the same lysine residue), heterotypic Ub chains (conjugation through different linkage patterns), branched chains, or monoubiquitination (38). Among the different types of polyubiquitin modifications, the principal and most abundant forms are K48-linked and K63-linked polyubiquitination. However, the outcomes of these different ubiquitination events for the substrate are distinct: K48-linked polyubiquitin chains are the best characterized and trigger substrates for proteasomal degradation more frequently than other modifications (139, 140), whereas K63-linked chains play non degradative roles in cellular signaling, intracellular trafficking, the DNA damage response, and other contexts (141, 142).

The K48- and K63-linked polyubiquitin modifications are also the most common types of PTMs identified in the proteins of the IFN-I signaling pathway (**Table 1**). Although many Ub E3 ligases responsible for the K48-linked ubiquitination of proteins have been identified over the years (22, 24, 143), the corresponding DUBs in antagonizing the degradation and maintaining the protein stability of the key molecules in IFN signaling remain poorly understood (144). An overall view of the DUBs that specifically hydrolyze K48-linked polyubiquitin chains from various substrates during virus infections such as CYLD, OTUD4, OTUD5, USP1, USP4, USP14, USP15, USP20, USP25, USP27X, USP29, and USP44 is summarized in **Table 1**. These DUBs specifically hydrolyze K48-linked polyubiquitin chains from various substrates and thereby stabilize proteins and play positive roles during viral infection. More specifically, among the DUBs, CYLD deficiency promotes K48-linked polyubiquitination and degradation of STING and thereby decreases the induction of IRF3-responsive genes after HSV-1 infection. In accord with this observation, CYLD-knockout mice are more susceptible to HSV-1 infection than their wild-type littermates (59). The deubiquitinase OTUD4 interacts with MAVS to remove its K48-linked polyubiquitin chains and thereby maintains MAVS stability and promotes innate antiviral signaling. Additionally, the knockout of OTUD4 impairs RNA virus-triggered activation of IRF3 and NF-κB and the expression of their downstream target genes, and potentiates VSV replication *in vitro* and *in vivo* (66). Similarly, OTUD5 promotes the protein stability of STING *via* cleaving the K48-linked polyubiquitin chains. The knockout of OTUD5 leads to faster turnover of STING and impairs IFN-I signaling following cytosolic DNA stimulation, whereas Lyz2-Cre Otud5^fl/Y^ mice and CD11-Cre Otud5^fl/Y^ mice show higher susceptibility to HSV-1 infection than their corresponding control littermates (69). Among the USP members, USP1 functions as a viral infection-induced physiological enhancer of TBK1 expression when bound to USP1 the K48-linked polyubiquitination of TBK1, resulting in enhanced TLR3/4 and RIG-I-induced IRF3 activation and IFNβ secretion (74). USP4, it positively regulates the RIG-I-mediated antiviral response by deubiquitinating K48-linked ubiquitin chains and stabilizing RIG-I (77). Interestingly, USP14, USP27X, and USP29 have been identified to positively regulate virus-induced IFN-I production by targeting the same substrate cGAS, and mechanistically, the three DUBs function by deubiquitinating K48-linked ubiquitin chains and stabilizing cGAS (83, 98, 99). Consistently, mice with the genetic ablation of USP27X and USP29 exhibit decreased levels of IFN-Is and proinflammatory cytokines after HSV-1 infection and hypersensitivity to HSV-1 infection compared with their wild-type littermates (98, 99). In addition, although both USP20 and USP44 have been shown to positively regulate virus-induced IFN-I signaling by targeting the same substrate, STING, and removing its K48-linked polyubiquitin chains, these two DUBs function differently (**Table 1**). Mechanistically, USP20 is recruited by USP18 to deconjugate K48-linked ubiquitination chains from STING and thus promotes the stability of STING and the expression of type I IFNs and proinflammatory cytokines after DNA virus infection (91). A later study, further confirmed that USP20 removes K48-linked ubiquitin chains from STING after HSV-1 infection and thereby stabilizes STING and promotes cellular antiviral responses (92). Congruently, USP20 knockout mice exhibit decreased levels of IFN-Is and proinflammatory cytokines, increased susceptibility to lethal HSV-1 infection, and aggravated HSV-1 replication compared with wild-type mice (92). The complementation of STING into Usp20 (-/-) cells remarkably restores HSV-1-triggered signaling and inhibits HSV-1 infection (92). In addition, the ectopic expression of USP15 enhances the TRIM25- and RIG-I-mediated production of type I IFN and thus suppresses RNA virus replication, whereas the depletion of USP15 causes decreased IFN production and markedly enhanced viral replication (85). Moreover, the DUB activity of USP25 is needed for virus-induced production of IFN-I and proinflammatory cytokines, because USP25 can stabilize TRAF3 by deubiquitinating K48-Ub on TRAF3 whereas the complemention of TRAF3/6 into USP25-deficient MEFs restores virus-induced signaling (96). Consistently, USP25-deficient mice are susceptible to H5N1 or HSV-1 infection than their wild-type counterparts (96).

Notably, although DUBs including OTUD1, USP5, and USP7 function to cleave K48-linked polyubiquitin chains of various substrates, the three DUBs exert opposite effects, which play negative roles in the host immune response against virus infection (**Table 1**). For example, OTUD1 upregulates the protein levels of intracellular Smurf1 by removing the K48-linked polyubiquitin chains of Smurf1, and RNA virus infection promotes the binding of Smurf1 to MAVS, TRAF3, and TRAF6, which leads to ubiquitination-dependent degradation of the three proteins and subsequent potent inhibition of IFNs production (64). In agreement with this observation, OTUD1-deficient mice produce more antiviral cytokines and are more resistant to RNA virus infection (64). In addition, a recent systematic functional screening assay revealed that USP5 inhibits IFNβ expression and promotes VSV replication by recruiting STIP1 homology and U-box containing protein 1 (STUB1) to degrade RIG-I (40). Whereas USP7 acts as a negative regulator in antiviral signaling by stabilizing TRIM27 and promoting the degradation of TBK1, the knockout of endogenous USP7 leads to enhanced TRIM27 degradation and reduced TBK1 ubiquitination and degradation (79). In the case of IFNAR-mediated downstream signaling pathway, USP2A sustains interferon antiviral activity by restricting the K48-linked ubiquitination of p-STAT1 in the nucleus (111). *Via* using RNA interference screening strategy, USP13 was found to positively regulate IFN-I signaling by deubiquitinating the K48-linked polyubiquitin chains the of STAT1 protein (115). Congruently, STAT1 ubiquitination is reduced in cells by USP13 overexpression and increased with USP13 knockdown regardless of IFNα treatment (115). JOSD1 has been identified to negatively regulate IFN-I-induced signaling and the antiviral response by deubiquitinating the K48-linked polyubiquitination of SOCS1, which is an essential negative regulator of many cytokine signaling pathways (120).

K63-linked polyubiquitin modification, it has also been identified as fundamental for both the innate and adaptive immune systems. K63-linked polyubiquitin is not only needed for the virus-induced activation of TBK1 and IRF3 (145) but also widely involved in pathways including NF-κB signaling and MAPK activation (146, 147). In NF-κB pathways, K63-linked polyubiquitin chains play pivotal roles in stabilizing the receptor signalosome on the membrane and hence facilitate the recruitment of adaptors or complexes and activating kinases (148). Critically, many E3 ligases, including TRAF6, are implicated in NF-κB pathways by catalyzing K63-linked polyubiquitination of various proteins (146). Whereas DUBs play an opposite role to E3 ligases, and various DUBs, including A20, CYLD, UCHL1, OTUD4, OTUD5, OTUD7B, USP18, USP25, and MYSM1, have been found to remove K63-linked polyubiquitin chains from various substrates (TBK1, TAK1, MyD88, TRAF3, and TRAF6) (**Table 1**). Intriguingly, unlike the aforementioned DUBs, A20 is a hybrid of a DUB and a E3 ligase and has an N-terminal OTU domain responsible for polyubiquitin cleavage and C-terminal domain-containing zinc fingers that bear E3 ligase activity. A20 cleaves the K63-linked polyubiquitin chains of TRAF6, RIP1, RIPK2, IKK-γ, and MALT1 and hence suppresses NF-κB activation. Moreover, A20 has been shown to promote the K48-linked ubiquitination of RIP1, which leads to its degradation and thereby the downregulation of NF-κB signaling (146, 149). Critically, K63-linked ubiquitination also plays a pivotal role in affecting virus-induced IFN-I production by either stabilizing substrates or by acting as a scaffold for the formation of a signaling multi complex (150). To date, a panel of 15 DUBs, such as CYLD, UCHL1, OTUD1, OTUD3, OTUD4, OTUD5, USP2B, USP3, USP14, USP15, USP21, USP25, USP27X, USP49, and MYSM1, have been identified to cleave the K63-linked polyubiquitin chains on various proteins, which results in a positive or negative effect on virus-induced IFN-I production under different contexts (**Table 1**). For example, OTUD3 removes K63-linked ubiquitin chains from MAVS and thereby inhibits MAVS aggregation and IFN-I signaling activation (65). In addition, unanchored K63 polyubiquitin chains can bind to MDA5, and this binding is important for signaling by MDA5, mutations of conserved residues in MDA5 disrupt its ubiquitin binding, and abrogate its ability to activate IRF3 (151). In the case of IFNAR1-mediated downstream signaling, BRCC36 sustains the protein turnover of IFNAR1 by removing K63-Ub from IFNAR1 (109), whereas USP5 has been identified to negatively regulate IFN-I-induced p-STAT1 activation and antiviral activities by removing K63-Ub on SMURF1 (112).

Additionally, some DUBs possess broad DUB activity against several types of Ub linkages. The DUBs OTUD7B, USP17, USP25, MCPIP1, ATXN3, and UCHL3 could simultaneously deconjugate the K48- and K63-linked Ub chains from the same protein in the IFN-I signaling pathway (**Table 1**). For instance, ATXN3 sustains IFNAR1-mediated downstream signaling by deubiquitinating both the K48- and K63-linked types of Ub chains on HDAC3 (108). However, USP13, USP19, and USP22 inhibit virus-induced IFN production by removing K27-linked polyubiquitin chains from STING (40, 81) or TRIF (90). In contrast, USP38 combines with the active form of TBK1 *via* the NLR family pyrin domain containing 4 (NLRP4) signalosome and then cleaves K33-linked Ub chains from TBK1 at Lys670, which allows DTX4 and TRIP to catalyze K48-linked ubiquitination on the same residue (101). This process causes the degradation of TBK1, thus negatively regulates IFN-I signaling. Intriguingly, USP39 promotes IFN-mediated antiviral responses by decreasing K6-linked but not canonical K48-linked polyubiquitination of STAT1 for degradation (118), eventhough K6-linked ubiquitin chains are often related to DNA damage instead of protein degradation (142). Morever, although USP5 reportedly increases K11- and K48-linked ubiquitination of RIG-I upon virus infection and thereby facilitates the degradation of RIG-I (40), the detailed mechanism used by USP5 to enhance K11-linked Ub chains of RIG-I and the exact functions of K11-linked Ub chains implicated in the RIG-I-mediated signaling pathway remain elusive. Overall, the atypical K6-, K11-, K27-, K33- and linear-linked polyubiquitin chains of proteins also play critical roles in antiviral immunity and inflammation (152). However, little is known about K29-linked polyubiquitination, and whether this type of PTM occurs on substrates involved in IFN-I signaling remains unknown and warrants further research.

## DUBs Regulate Host Antiviral Activity Independently of Their Protease Activity

Although many studies have demonstrated that the protease activity of DUBs is critical in regulating the Ub chains on their substrates and affecting host IFN immune responses, some studies have also shown that the catalytic activity of certain DUBs is not necessary in regulating the IFN-I signaling pathway, which implies novel strategies used by DUBs. Mechanistically, the catalytically inactive mutant sites of DUBs could not abolish their negative or positive roles during virus infection, which indicates that these DUBs function independently of their protease activity. For instance, both the wild-type and enzymatically inactive mutant of USP5 can cause a decreased polyubiquitination level of SMURF1 (112), which suggest that USP5 functions in the immune response probably independently of its protease activity. In addition, some DUBs form complexes with adaptor or scaffold proteins, which act by recruiting proteins to participate in particular biological events, attracting trafficking factors that change substrate localization, or controlling substrate activity. For instance, DUBs can regulate a specific substrate by recruiting other factors, as demonstrated by USP10 recruits and binds with monocyte chemotactic protein induced protein 1 (MCPIP1) to deubiquitinate its substrate, nuclear factor κB essential modulator (NEMO) (153). Additionally, it has been shown that A20 blocks antiviral signaling by disrupting K63-linked polyubiquitination of TBK1-IKK complex independtly of the A20 deubiquitination domain (154). Futhermore, A20 prevents the interaction between Ubc13 and both TRAF2/5 and cIAP1/2 upon TNFα stimulation, which suggest A20 functions beyond its protease activity (155). In addition, A20 suppresses TNFα-induced NF-κB signaling through a noncatalytic mechanism that involves binding to polyubiquitin chains *via* its seventh zinc finger (ZnF7) (56, 156, 157). This binding is proposed to impede the recruitment of other linear polyubiquitin binding proteins that are essential for productive signaling downstream from TNFR (157). Moreover, USP5 suppresses IFN-β expression and enhances VSV replication by recruiting STUB1 to degrade RIG-I (40). USP13, which shares ∼80% sequence similarity with USP5, negatively regulates virus-induced IFN-I production by inhibiting the recruitment of TBK1 to STING by deubiquitinating the K27-linked ubiquitin chains on STING (81), whereas USP22 recruits USP13 to cleave the K27-linked polyubiquitin chains from STING (40). USP18 does not deubiquitinate STING *in vitro* but facilitates USP20 to catalyze deubiquitination of STING in a manner independently of the enzymatic activity of USP18 (91). In addition, USP18-knockout mice are more susceptible to HSV-1 infection than their wild-type littermates, and the reintroduction of STING into USP18^−/−^ MEFs can restore the HSV-1-induced expression of downstream genes and cellular antiviral responses (91). In addition to being an active enzyme, USP18 can bind to the intracellular part of IFNAR2 and compete with the binding of JAK1 to the receptor, which results in negative regulation of IFNAR signaling independently of its protease activity (117). In the case of IFNAR-mediated downstream signaling, some other DUBs also implement their functions beyond their protease activities. For example, BRCC36 functions noncatalytically by recruiting USP13 to counteract the SMURF1-mediated degradation of STAT1, and this effect enhances the stability of STAT1 and improves host antiviral efficiency (110). Additionally, USP12 positively regulates IFN antiviral signaling independently of its deubiquitinase activity. Upon IFN treatment, USP12 accumulates in the nucleus, blocks the CREB-binding protein-induced acetylation of p-STAT1, and thus inhibits the dephosphorylation effects of TCPTP on p-STAT1, which ultimately maintains the nuclear p-STAT1 levels and IFN antiviral efficacy (114).

## DUB Inhibitors and Their Potential Roles in Therapeutic Purposes

Because DUBs play critical roles during innate antiviral responses, the development of small-molecule inhibitors that specifically change DUB activities might be a therapeutic strategy for improving host antiviral efficiency. Over the years, inhibitors of a panel of DUBs, including USP1, USP2, USP4, USP5, USP7, USP8, USP9X, USP10, USP11, USP13, USP14, USP19, USP20, USP25/28, USP30, COPS5, STAMBP, PSMD14, UCHL1, UCHL3 and UCHL5, have been designed (158–163). However, to date, only a few small-molecule inhibitors of DUBs have been employed to investigate their functional roles in host antiviral activities. For instance, the USP7 inhibitors P5091 and P22077 have been verified to promote the type-I interferon-mediated antiviral response by destabilizing SOCS1 (113). Similarly, the USP5 inhibitor PYR41 could reduce virus replication at the mRNA and protein levels by promoting IFNAR-mediated antiviral responses (112).

Because ubiquitination and related processes are involved in myriad aspects of human cell biology and physiology, abnormalities in such events can cause many diseases. Among these events, the dysregulation of DUB activity contributes to various sporadic and genetic diseases (158, 164, 165). For instance, human USP18 deficiency underlies type 1 interferonopathy, leading to severe pseudo-TORCH syndrome which is characterized by microcephaly, enlarged ventricles, cerebral calcification, and other severe complications (166). Similarly, the homozygous mutation of USP18 also causes severe type I interferonopathy because the mutated USP18 protein results in unmitigated interferon-mediated inflammation and is lethal during the perinatal period (167). However, the treatment of these patients with ruxolitinib, a JAK 1/2 inhibitor, it significantly improves their symptoms (167). Additionally, a homozygous miss-sense mutation in STAT2 results in failure to appropriately traffic USP18 to IFNAR2 and prevents USP18 from negatively regulating responses to IFN-Is, which leads to infant death from autoinflammation disease (168). Notably, given that the current therapeutics remains incapable of achieving satisfying disease management in all patients, the therapeutic modulation of DUBs might be an attractive target in certain diseases. As has been demonstrated, although some inhibitors can treat cancer disease efficiently (169), the use of these inhibitors in the treatment of viral infectious diseases remains largely unexplored. Because DUB inhibition could promote steady-state Ub levels of specific substrates without affecting global protein or Ub levels, the development of small-molecule inhibitors targeted towards DUBs has increasingly become a promising strategy for drug discovery (170). However, because many DUBs are conserved during evolution and have a high sequence similarity, new perspectives are needed to facilitate the development of specific inhibitors. Consequently, the design of small-molecule inhibitors that interfere with the activity of DUBs or the DUB-substrate interactions accompanied by their relevance *in vivo* and related diseases remains one of the critical and challenging research areas.

## Conclusions and Perspectives

In summary, DUB-mediated regulation represents a crucial mechanism used by hosts to tightly regulate the extent of IFN signaling to achieve a balance between pathogen eradication and the prevention of excessive immune responses. However, how DUBs implement their diverse functions and interact with substrates in a dynamic, temporal, and spatial manner to ensure the most favourable outcome remains elusive. Intriguingly, some viruses also encode DUBs and other proteins that either act alone or interact with other cellular components to evade host immune surveillance (171, 172). Thus, the interplay between DUBs and pathogens might add a new sophisticated mchanism that regulates the timing and amplitude of host immune responses to viral challenges. In addition, how PTMs (such as phosphorylation, acetylation, and methylation) of DUBs and, Ub and other unconventional Ub structures modulate the functional shift of DUBs and thus affect host innate immune signaling, is still poorly understood. Future studies exploring the detailed mechanisms of DUBs, their inducers, and downstream targets during viral infections might help improve the present understanding of the mechanisms of host innate immune responses, and these findings could lead to the identification of novel targets and help guide the development of therapeutic strategies for the treatment of human diseases.

## Author Contributions

The table and graphs were prepared by LZ. The literature was collected and analyzed by GL and YL. JP and ZZ provided insightful comments and suggestions on the manuscript. GQ and HL wrote the paper and critically revised the draft. All authors contributed to the article and approved the submitted version.

## Funding

Work in our laboratory is supported by grants from the National Natural Science Foundation of China (No. 31800982, No. 81971477, No. 82171797, No. 81870365, No. 82070512, No. 81702737, No. 81803116 and No. 81902534), Jiangsu Provincial Medical Young Talents (QNRC2016756), the Applied Foundational Research of Medical and Health Care of Suzhou City (SYS2019086, SYS2019083), and Gusu Health Talent Program (GSWS2020038).

## Conflict of Interest

The authors declare that the research was conducted in the absence of any commercial or financial relationships that could be construed as a potential conflict of interest.

## Publisher’s Note

All claims expressed in this article are solely those of the authors and do not necessarily represent those of their affiliated organizations, or those of the publisher, the editors and the reviewers. Any product that may be evaluated in this article, or claim that may be made by its manufacturer, is not guaranteed or endorsed by the publisher.

## References

[1] Hadjadj J Yatim N Barnabei L Corneau A Boussier J Smith N. Impaired Type I Interferon Activity and Inflammatory Responses in Severe COVID-19 Patients. Science (2020) 369(6504):718–24. doi: 10.1126/science.abc6027 PMC7402632 32661059

[2] Brubaker SW Bonham KS Zanoni I Kagan JC. Innate Immune Pattern Recognition: A Cell Biological Perspective. Annu Rev Immunol (2015) 33:257–90. doi: 10.1146/annurev-immunol-032414-112240 PMC5146691 25581309

[3] Carty M Guy C Bowie AG. Detection of Viral Infections by Innate Immunity. Biochem Pharmacol (2021) 183:114316. doi: 10.1016/j.bcp.2020.114316 33152343

[4] Wu J Chen ZJ. Innate Immune Sensing and Signaling of Cytosolic Nucleic Acids. Annu Rev Immunol (2014) 32:461–88. doi: 10.1146/annurev-immunol-032713-120156 24655297

[5] Heaton SM Borg NA Dixit VM. Ubiquitin in the Activation and Attenuation of Innate Antiviral Immunity. J Exp Med (2016) 213(1):1–13. doi: 10.1084/jem.20151531 26712804 PMC4710203

[6] Barrat FJ Elkon KB Fitzgerald KA. Importance of Nucleic Acid Recognition in Inflammation and Autoimmunity. Annu Rev Med (2016) 67:323–36. doi: 10.1146/annurev-med-052814-023338 26526766

[7] Takeuchi O Akira S. Pattern Recognition Receptors and Inflammation. Cell (2010) 140(6):805–20. doi: 10.1016/j.cell.2010.01.022 20303872

[8] Honda K Ohba Y Yanai H Negishi H Mizutani T Takaoka A. Spatiotemporal Regulation of MyD88-IRF-7 Signalling for Robust Type-I Interferon Induction. Nature (2005) 434(7036):1035–40. doi: 10.1038/nature03547 15815647

[9] Wang L Zhao J Ren J Hall KH Moorman JP Yao ZQ. Protein Phosphatase 1 Abrogates IRF7-Mediated Type I IFN Response in Antiviral Immunity. Eur J Immunol (2016) 46(10):2409–19. doi: 10.1002/eji.201646491 PMC5175453 27469204

[10] Hartmann G. Nucleic Acid Immunity. Adv Immunol (2017) 133:121–69. doi: 10.1016/bs.ai.2016.11.001 PMC7112058 28215278

[11] Rehwinkel J Gack MU. RIG-I-Like Receptors: Their Regulation and Roles in RNA Sensing. Nat Rev Immunol (2020) 20(9):537–51. doi: 10.1038/s41577-020-0288-3 PMC7094958 32203325

[12] Kopitar-Jerala N. The Role of Interferons in Inflammation and Inflammasome Activation. Front Immunol (2017) 8:873. doi: 10.3389/fimmu.2017.00873 28791024 PMC5525294

[13] Fuchs SY. Ubiquitination-Mediated Regulation of Interferon Responses. Growth Factors (2012) 30(3):141–8. doi: 10.3109/08977194.2012.669382 PMC3703147 22394219

[14] Borden EC Sen GC Uze G Silverman RH Ransohoff RM Foster GR. Interferons at Age 50: Past, Current and Future Impact on Biomedicine. Nat Rev Drug Discovery (2007) 6(12):975–90. doi: 10.1038/nrd2422 PMC7097588 18049472

[15] Pestka S Krause CD Walter MR. Interferons, Interferon-Like Cytokines, and Their Receptors. Immunol Rev (2004) 202:8–32. doi: 10.1111/j.0105-2896.2004.00204.x 15546383

[16] Crow MK Olferiev M Kirou KA. Type I Interferons in Autoimmune Disease. Annu Rev Pathol (2019) 14:369–93. doi: 10.1146/annurev-pathol-020117-043952 30332560

[17] McNab F Mayer-Barber K Sher A Wack A O’Garra A. Type I Interferons in Infectious Disease. Nat Rev Immunol (2015) 15(2):87–103. doi: 10.1038/nri3787 25614319 PMC7162685

[18] Chen X Saccon E Appelberg KS Mikaeloff F Rodriguez JE Vinhas BS. Type-I Interferon Signatures in SARS-CoV-2 Infected Huh7 Cells. Cell Death Discov (2021) 7(1):114. doi: 10.1038/s41420-021-00487-z 34006825 PMC8129603

[19] Cheng Z Dai T He X Zhang Z Xie F Wang S. The Interactions Between cGAS-STING Pathway and Pathogens. Signal Transduct Target Ther (2020) 5(1):91. doi: 10.1038/s41392-020-0198-7 32532954 PMC7293265

[20] Honda K Takaoka A Taniguchi T. Type I Interferon [Corrected] Gene Induction by the Interferon Regulatory Factor Family of Transcription Factors. Immunity (2006) 25(3):349–60. doi: 10.1016/j.immuni.2006.08.009 16979567

[21] Sadler AJ Williams BR. Interferon-Inducible Antiviral Effectors. Nat Rev Immunol (2008) 8(7):559–68. doi: 10.1038/nri2314 PMC2522268 18575461

[22] Liu J Qian C Cao X. Post-Translational Modification Control of Innate Immunity. Immunity (2016) 45(1):15–30. doi: 10.1016/j.immuni.2016.06.020 27438764

[23] Zhou Y He C Wang L Ge B. Post-Translational Regulation of Antiviral Innate Signaling. Eur J Immunol (2017) 47(9):1414–26. doi: 10.1002/eji.201746959 PMC7163624 28744851

[24] Davis ME Gack MU. Ubiquitination in the Antiviral Immune Response. Virology (2015) 479-480:52–65. doi: 10.1016/j.virol.2015.02.033 25753787 PMC4774549

[25] Ciehanover A Hod Y. Hershko A. A Heat-Stable Polypeptide Component of an ATP-Dependent Proteolytic System From Reticulocytes. Biochem Biophys Res Commun (1978) 81(4):1100–5. doi: 10.1016/0006-291x(78)91249-4 666810

[26] Hershko A Ciechanover A. The Ubiquitin System. Annu Rev Biochem (1998) 67:425–79. doi: 10.1146/annurev.biochem.67.1.425 9759494

[27] Komander D Rape M. The Ubiquitin Code. Annu Rev Biochem (2012) 81:203–29. doi: 10.1146/annurev-biochem-060310-170328 22524316

[28] Ohtake F Tsuchiya H. The Emerging Complexity of Ubiquitin Architecture. J Biochem (2017) 161(2):125–33. doi: 10.1093/jb/mvw088 28011818

[29] Yau R Rape M. The Increasing Complexity of the Ubiquitin Code. Nat Cell Biol (2016) 18(6):579–86. doi: 10.1038/ncb3358 27230526

[30] Kirisako T Kamei K Murata S Kato M Fukumoto H Kanie M. A Ubiquitin Ligase Complex Assembles Linear Polyubiquitin Chains. EMBO J (2006) 25(20):4877–87. doi: 10.1038/sj.emboj.7601360 PMC1618115 17006537

[31] Haakonsen DL Rape M. Branching Out: Improved Signaling by Heterotypic Ubiquitin Chains. Trends Cell Biol (2019) 29(9):704–16. doi: 10.1016/j.tcb.2019.06.003 31300189

[32] Coleman KE Huang TT. In a Class of Its Own: A New Family of Deubiquitinases Promotes Genome Stability. Mol Cell (2018) 70(1):1–3. doi: 10.1016/j.molcel.2018.03.022 29625031

[33] Matsushita K Takeuchi O Standley DM Kumagai Y Kawagoe T Miyake T. Zc3h12a Is an RNase Essential for Controlling Immune Responses by Regulating mRNA Decay. Nature (2009) 458(7242):1185–90. doi: 10.1038/nature07924 19322177

[34] Xie X Wang X Jiang D Wang J Fei R Cong X. PPPDE1 is a Novel Deubiquitinase Belonging to a Cysteine Isopeptidase Family. Biochem Biophys Res Commun (2017) 488(2):291–6. doi: 10.1016/j.bbrc.2017.04.161 28483520

[35] Kwasna D Abdul Rehman SA Natarajan J Matthews S Madden R De Cesare V. Discovery and Characterization of ZUFSP/ZUP1, a Distinct Deubiquitinase Class Important for Genome Stability. Mol Cell (2018) 70(1):150–64 e6. doi: 10.1016/j.molcel.2018.02.023 29576527 PMC5896202

[36] Abdul Rehman SA Kristariyanto YA Choi SY Nkosi PJ Weidlich S Labib K. MINDY-1 Is a Member of an Evolutionarily Conserved and Structurally Distinct New Family of Deubiquitinating Enzymes. Mol Cell (2016) 63(1):146–55. doi: 10.1016/j.molcel.2016.05.009 PMC4942677 27292798

[37] Nijman SM Luna-Vargas MP Velds A Brummelkamp TR Dirac AM Sixma TK. A Genomic and Functional Inventory of Deubiquitinating Enzymes. Cell (2005) 123(5):773–86. doi: 10.1016/j.cell.2005.11.007 16325574

[38] Reyes-Turcu FE Wilkinson KD. Polyubiquitin Binding and Disassembly by Deubiquitinating Enzymes. Chem Rev (2009) 109(4):1495–508. doi: 10.1021/cr800470j PMC2734106 19243136

[39] Mevissen TET Komander D. Mechanisms of Deubiquitinase Specificity and Regulation. Annu Rev Biochem (2017) 86:159–92. doi: 10.1146/annurev-biochem-061516-044916 28498721

[40] Liu Q Wu Y Qin Y Hu J Xie W Qin FX-F. Broad and Diverse Mechanisms Used by Deubiquitinase Family Members in Regulating the Type I Interferon Signaling Pathway During Antiviral Responses. Sci Adv (2018) 4(5):eaar2824. doi: 10.1126/sciadv.aar2824 29732405 PMC5931765

[41] Bhoj VG Chen ZJ. Ubiquitylation in Innate and Adaptive Immunity. Nature (2009) 458(7237):430–7. doi: 10.1038/nature07959 19325622

[42] Kowalinski E Lunardi T McCarthy AA Louber J Brunel J Grigorov B. Structural Basis for the Activation of Innate Immune Pattern-Recognition Receptor RIG-I by Viral RNA. Cell (2011) 147(2):423–35. doi: 10.1016/j.cell.2011.09.039 22000019

[43] Kato H Sato S Yoneyama M Yamamoto M Uematsu S Matsui K. Cell Type-Specific Involvement of RIG-I in Antiviral Response. Immunity (2005) 23(1):19–28. doi: 10.1016/j.immuni.2005.04.010 16039576

[44] Kato H Takeuchi O Sato S Yoneyama M Yamamoto M Matsui K. Differential Roles of MDA5 and RIG-I Helicases in the Recognition of RNA Viruses. Nature (2006) 441(7089):101–5. doi: 10.1038/nature04734 16625202

[45] Gack MU Shin YC Joo CH Urano T Liang C Sun L. TRIM25 RING-Finger E3 Ubiquitin Ligase Is Essential for RIG-I-Mediated Antiviral Activity. Nature (2007) 446(7138):916–20. doi: 10.1038/nature05732 17392790

[46] Oshiumi H Matsumoto M Hatakeyama S Seya T. Riplet/RNF135, a RING Finger Protein, Ubiquitinates RIG-I to Promote Interferon-Beta Induction During the Early Phase of Viral Infection. J Biol Chem (2009) 284(2):807–17. doi: 10.1074/jbc.M804259200 19017631

[47] Arimoto K Takahashi H Hishiki T Konishi H Fujita T Shimotohno K. Negative Regulation of the RIG-I Signaling by the Ubiquitin Ligase RNF125. Proc Natl Acad Sci USA (2007) 104(18):7500–5. doi: 10.1073/pnas.0611551104 PMC1863485 17460044

[48] Wang W Jiang M Liu S Zhang S Liu W Ma Y. RNF122 Suppresses Antiviral Type I Interferon Production by Targeting RIG-I CARDs to Mediate RIG-I Degradation. Proc Natl Acad Sci USA (2016) 113(34):9581–6. doi: 10.1073/pnas.1604277113 PMC5003265 27506794

[49] Zhao C Jia M Song H Yu Z Wang W Li Q. The E3 Ubiquitin Ligase TRIM40 Attenuates Antiviral Immune Responses by Targeting MDA5 and RIG-I. Cell Rep (2017) 21(6):1613–23. doi: 10.1016/j.celrep.2017.10.020 29117565

[50] Zhou P Ding X Wan X Liu L Yuan X Zhang W. MLL5 Suppresses Antiviral Innate Immune Response by Facilitating STUB1-Mediated RIG-I Degradation. Nat Commun (2018) 9(1):1243. doi: 10.1038/s41467-018-03563-8 29593341 PMC5871759

[51] Chen W Han C Xie B Hu X Yu Q Shi L. Induction of Siglec-G by RNA Viruses Inhibits the Innate Immune Response by Promoting RIG-I Degradation. Cell (2013) 152(3):467–78. doi: 10.1016/j.cell.2013.01.011 23374343

[52] Okamoto M Kouwaki T Fukushima Y Oshiumi H. Regulation of RIG-I Activation by K63-Linked Polyubiquitination. Front Immunol (2017) 8:1942. doi: 10.3389/fimmu.2017.01942 29354136 PMC5760545

[53] Lin R Yang L Nakhaei P Sun Q Sharif-Askari E Julkunen I. Negative Regulation of the Retinoic Acid-Inducible Gene I-Induced Antiviral State by the Ubiquitin-Editing Protein A20. J Biol Chem (2006) 281(4):2095–103. doi: 10.1074/jbc.M510326200 16306043

[54] Ning S Pagano JS. The A20 Deubiquitinase Activity Negatively Regulates LMP1 Activation of IRF7. J Virol (2010) 84(12):6130–8. doi: 10.1128/JVI.00364-10 PMC2876664 20392859

[55] Boone DL Turer EE Lee EG Ahmad RC Wheeler MT Tsui C. The Ubiquitin-Modifying Enzyme A20 Is Required for Termination of Toll-Like Receptor Responses. Nat Immunol (2004) 5(10):1052–60. doi: 10.1038/ni1110 15334086

[56] Skaug B Chen J Du F He J Ma A Chen ZJ. Direct, Noncatalytic Mechanism of IKK Inhibition by A20. Mol Cell (2011) 44(4):559–71. doi: 10.1016/j.molcel.2011.09.015 PMC3237303 22099304

[57] Zhang J Stirling B Temmerman ST Ma CA Fuss IJ Derry JM. Impaired Regulation of NF-kappaB and Increased Susceptibility to Colitis-Associated Tumorigenesis in CYLD-Deficient Mice. J Clin Invest (2006) 116(11):3042–9. doi: 10.1172/JCI28746 PMC1616194 17053834

[58] Friedman CS O’Donnell MA Legarda-Addison D Ng A Cardenas WB Yount JS. The Tumour Suppressor CYLD Is a Negative Regulator of RIG-I-Mediated Antiviral Response. EMBO Rep (2008) 9(9):930–6. doi: 10.1038/embor.2008.136 PMC2529351 18636086

[59] Zhang L Wei N Cui Y Hong Z Liu X Wang Q. The Deubiquitinase CYLD Is a Specific Checkpoint of the STING Antiviral Signaling Pathway. PloS Pathog (2018) 14(11):e1007435. doi: 10.1371/journal.ppat.1007435 30388174 PMC6235404

[60] Karim R Tummers B Meyers C Biryukov JL Alam S Backendorf C. Human Papillomavirus (HPV) Upregulates the Cellular Deubiquitinase UCHL1 to Suppress the Keratinocyte’s Innate Immune Response. PloS Pathog (2013) 9(5):e1003384. doi: 10.1371/journal.ppat.1003384 23717208 PMC3662672

[61] Li S Zheng H Mao AP Zhong B Li Y Liu Y. Regulation of Virus-Triggered Signaling by OTUB1- and OTUB2-Mediated Deubiquitination of TRAF3 and TRAF6. J Biol Chem (2010) 285(7):4291–7. doi: 10.1074/jbc.M109.074971 PMC2836033 19996094

[62] Lu D Song J Sun Y Qi F Liu L Jin Y. Mutations of Deubiquitinase OTUD1 Are Associated With Autoimmune Disorders. J Autoimmun (2018) 94:156–65. doi: 10.1016/j.jaut.2018.07.019 30100102

[63] Zhang Z Wang D Wang P Zhao Y You F. OTUD1 Negatively Regulates Type I IFN Induction by Disrupting Noncanonical Ubiquitination of IRF3. J Immunol (2020) 204(7):1904–18. doi: 10.4049/jimmunol.1900305 32075857

[64] Zhang L Liu J Qian L Feng Q Wang X Yuan Y. Induction of OTUD1 by RNA Viruses Potently Inhibits Innate Immune Responses by Promoting Degradation of the MAVS/TRAF3/TRAF6 Signalosome. PloS Pathog (2018) 14(5):e1007067. doi: 10.1371/journal.ppat.1007067 29734366 PMC5957451

[65] Zhang Z Fang X Wu X Ling L Chu F Li J. Acetylation-Dependent Deubiquitinase OTUD3 Controls MAVS Activation in Innate Antiviral Immunity. Mol Cell (2020) 79(2):304–19.e7. doi: 10.1016/j.molcel.2020.06.020 32679077

[66] Liuyu T Yu K Ye L Zhang Z Zhang M Ren Y. Induction of OTUD4 by Viral Infection Promotes Antiviral Responses Through Deubiquitinating and Stabilizing MAVS. Cell Res (2019) 29(1):67–79. doi: 10.1038/s41422-018-0107-6 30410068 PMC6318273

[67] Zhao Y Mudge MC Soll JM Rodrigues RB Byrum AK Schwarzkopf EA. OTUD4 Is a Phospho-Activated K63 Deubiquitinase That Regulates MyD88-Dependent Signaling. Mol Cell (2018) 69(3):505–16.e5. doi: 10.1016/j.molcel.2018.01.009 29395066 PMC6819006

[68] Kayagaki N Phung Q Chan S Chaudhari R Quan C O’Rourke KM. DUBA: A Deubiquitinase That Regulates Type I Interferon Production. Science (2007) 318(5856):1628–32. doi: 10.1126/science.1145918 17991829

[69] Guo Y Jiang F Kong L Wu H Zhang H Chen X. OTUD5 Promotes Innate Antiviral and Antitumor Immunity Through Deubiquitinating and Stabilizing STING. Cell Mol Immunol (2020) 18(8):1945–55. doi: 10.1038/s41423-020-00531-5 PMC8322343 32879469

[70] Enesa K Zakkar M Chaudhury H Luong le A Rawlinson L Mason JC. NF-kappaB Suppression by the Deubiquitinating Enzyme Cezanne: A Novel Negative Feedback Loop in Pro-Inflammatory Signaling. J Biol Chem (2008) 283(11):7036–45. doi: 10.1074/jbc.M708690200 18178551

[71] Ji Y Cao L Zeng L Zhang Z Xiao Q Guan P. The N-Terminal Ubiquitin-Associated Domain of Cezanne Is Crucial for Its Function to Suppress NF-kappaB Pathway. J Cell Biochem (2018) 119(2):1979–91. doi: 10.1002/jcb.26359 28817177

[72] Hu H Brittain GC Chang JH Puebla-Osorio N Jin J Zal A. OTUD7B Controls Non-Canonical NF-kappaB Activation Through Deubiquitination of TRAF3. Nature (2013) 494(7437):371–4. doi: 10.1038/nature11831 PMC3578967 23334419

[73] Luong le A Fragiadaki M Smith J Boyle J Lutz J Dean JL. Cezanne Regulates Inflammatory Responses to Hypoxia in Endothelial Cells by Targeting TRAF6 for Deubiquitination. Circ Res (2013) 112(12):1583–91. doi: 10.1161/CIRCRESAHA.111.300119 PMC7611065 23564640

[74] Yu Z Song H Jia M Zhang J Wang W Li Q. USP1-UAF1 Deubiquitinase Complex Stabilizes TBK1 and Enhances Antiviral Responses. J Exp Med (2017) 214(12):3553–63. doi: 10.1084/jem.20170180 PMC5716033 29138248

[75] Zhang L Zhao X Zhang M Zhao W Gao C. Ubiquitin-Specific Protease 2b Negatively Regulates IFN-Beta Production and Antiviral Activity by Targeting TANK-Binding Kinase 1. J Immunol (2014) 193(5):2230–7. doi: 10.4049/jimmunol.1302634 25070846

[76] Cui J Song Y Li Y Zhu Q Tan P Qin Y. USP3 Inhibits Type I Interferon Signaling by Deubiquitinating RIG-I-Like Receptors. Cell Res (2014) 24(4):400–16. doi: 10.1038/cr.2013.170 PMC3975496 24366338

[77] Wang L Zhao W Zhang M Wang P Zhao K Zhao X. USP4 Positively Regulates RIG-I-Mediated Antiviral Response Through Deubiquitination and Stabilization of RIG-I. J Virol (2013) 87(8):4507–15. doi: 10.1128/JVI.00031-13 PMC3624380 23388719

[78] Xu C Peng Y Zhang Q Xu XP Kong XM Shi WF. USP4 Positively Regulates RLR-Induced NF-kappaB Activation by Targeting TRAF6 for K48-Linked Deubiquitination and Inhibits Enterovirus 71 Replication. Sci Rep (2018) 8(1):13418. doi: 10.1038/s41598-018-31734-6 30194441 PMC6128947

[79] Cai J Chen HY Peng SJ Meng JL Wang Y Zhou Y. USP7-TRIM27 Axis Negatively Modulates Antiviral Type I IFN Signaling. FASEB J: Off Publ Fed Am Soc Exp Biol (2018) 32(10):5238–49. doi: 10.1096/fj.201700473RR 29688809

[80] Colleran A Collins PE O’Carroll C Ahmed A Mao X McManus B. Deubiquitination of NF-kappaB by Ubiquitin-Specific Protease-7 Promotes Transcription. Proc Natl Acad Sci USA (2013) 110(2):618–23. doi: 10.1073/pnas.1208446110 PMC3545798 23267096

[81] Sun H Zhang Q Jing YY Zhang M Wang HY Cai Z. USP13 Negatively Regulates Antiviral Responses by Deubiquitinating STING. Nat Commun (2017) 8:15534. doi: 10.1038/ncomms15534 28534493 PMC5457515

[82] Li H Zhao Z Ling J Pan L Zhao X Zhu H. USP14 Promotes K63-Linked RIG-I Deubiquitination and Suppresses Antiviral Immune Responses. Eur J Immunol (2019) 49(1):42–53. doi: 10.1002/eji.201847603 30466171

[83] Chen M Meng Q Qin Y Liang P Tan P He L. TRIM14 Inhibits cGAS Degradation Mediated by Selective Autophagy Receptor P62 to Promote Innate Immune Responses. Mol Cell (2016) 64(1):105–19. doi: 10.1016/j.molcel.2016.08.025 27666593

[84] Zhang H Wang D Zhong H Luo R Shang M Liu D. Ubiquitin-Specific Protease 15 Negatively Regulates Virus-Induced Type I Interferon Signaling *via* Catalytically-Dependent and -Independent Mechanisms. Sci Rep (2015) 5:11220. doi: 10.1038/srep11220 26061460 PMC4650652

[85] Pauli EK Chan YK Davis ME Gableske S Wang MK Feister KF. The Ubiquitin-Specific Protease USP15 Promotes RIG-I-Mediated Antiviral Signaling by Deubiquitylating TRIM25. Sci Signal (2014) 7(307):ra3. doi: 10.1126/scisignal.2004577 24399297 PMC4008495

[86] Torre S Polyak MJ Langlais D Fodil N Kennedy JM Radovanovic I. USP15 Regulates Type I Interferon Response and Is Required for Pathogenesis of Neuroinflammation. Nat Immunol (2017) 18(1):54–63. doi: 10.1038/ni.3581 27721430

[87] Chen R Zhang L Zhong B Tan B Liu Y Shu HB. The Ubiquitin-Specific Protease 17 Is Involved in Virus-Triggered Type I IFN Signaling. Cell Res (2010) 20(7):802–11. doi: 10.1038/cr.2010.41 20368735

[88] Ritchie KJ Hahn CS Kim KI Yan M Rosario D Li L. Role of ISG15 Protease UBP43 (USP18) in Innate Immunity to Viral Infection. Nat Med (2004) 10(12):1374–8. doi: 10.1038/nm1133 15531891

[89] Yang Z Xian H Hu J Tian S Qin Y Wang RF. USP18 Negatively Regulates NF-kappaB Signaling by Targeting TAK1 and NEMO for Deubiquitination Through Distinct Mechanisms. Sci Rep (2015) 5:12738. doi: 10.1038/srep12738 26240016 PMC4523862

[90] Wu X Lei C Xia T Zhong X Yang Q Shu HB. Regulation of TRIF-Mediated Innate Immune Response by K27-Linked Polyubiquitination and Deubiquitination. Nat Commun (2019) 10(1):4115. doi: 10.1038/s41467-019-12145-1 31511519 PMC6739404

[91] Zhang M Zhang MX Zhang Q Zhu GF Yuan L Zhang DE. USP18 Recruits USP20 to Promote Innate Antiviral Response Through Deubiquitinating STING/MITA. Cell Res (2016) 26(12):1302–19. doi: 10.1038/cr.2016.125 PMC5143414 27801882

[92] Zhang MX Cai Z Zhang M Wang XM Wang Y Zhao F. USP20 Promotes Cellular Antiviral Responses *via* Deconjugating K48-Linked Ubiquitination of MITA. J Immunol (2019) 202(8):2397–406. doi: 10.4049/jimmunol.1801447 30814308

[93] Fan Y Mao R Yu Y Liu S Shi Z Cheng J. USP21 Negatively Regulates Antiviral Response by Acting as a RIG-I Deubiquitinase. J Exp Med (2014) 211(2):313–28. doi: 10.1084/jem.20122844 PMC3920558 24493797

[94] Cai Z Zhang MX Tang Z Zhang Q Ye J Xiong TC. USP22 Promotes IRF3 Nuclear Translocation and Antiviral Responses by Deubiquitinating the Importin Protein KPNA2. J Exp Med (2020) 217(5):1–19. doi: 10.1084/jem.20191174 PMC7201923 32130408

[95] Zhong H Wang D Fang L Zhang H Luo R Shang M. Ubiquitin-Specific Proteases 25 Negatively Regulates Virus-Induced Type I Interferon Signaling. PloS One (2013) 8(11):e80976. doi: 10.1371/journal.pone.0080976 24260525 PMC3832446

[96] Lin D Zhang M Zhang MX Ren Y Jin J Zhao Q. Induction of USP25 by Viral Infection Promotes Innate Antiviral Responses by Mediating the Stabilization of TRAF3 and TRAF6. Proc Natl Acad Sci USA (2015) 112(36):11324–9. doi: 10.1073/pnas.1509968112 PMC4568686 26305951

[97] Tao X Chu B Xin D Li L Sun Q. USP27X Negatively Regulates Antiviral Signaling by Deubiquitinating RIG-I. PloS Pathog (2020) 16(2):e1008293. doi: 10.1371/journal.ppat.1008293 32027733 PMC7029883

[98] Guo Y Jiang F Kong L Li B Yang Y Zhang L. Cutting Edge: USP27X Deubiquitinates and Stabilizes the DNA Sensor cGAS to Regulate Cytosolic DNA-Mediated Signaling. J Immunol (2019) 203(8):2049–54. doi: 10.4049/jimmunol.1900514 31534008

[99] Zhang Q Tang Z An R Ye L Zhong B. USP29 Maintains the Stability of cGAS and Promotes Cellular Antiviral Responses and Autoimmunity. Cell Res (2020) 30(10):914–27. doi: 10.1038/s41422-020-0341-6 PMC7608407 32457395

[100] Li S Wang D Zhao J Weathington NM Shang D Zhao Y. The Deubiquitinating Enzyme USP48 Stabilizes TRAF2 and Reduces E-Cadherin-Mediated Adherens Junctions. FASEB J: Off Publ Fed Am Soc Exp Biol (2018) 32(1):230–42. doi: 10.1096/fj.201700415RR PMC5731130 28874458

[101] Lin M Zhao Z Yang Z Meng Q Tan P Xie W. USP38 Inhibits Type I Interferon Signaling by Editing TBK1 Ubiquitination Through NLRP4 Signalosome. Mol Cell (2016) 64(2):267–81. doi: 10.1016/j.molcel.2016.08.029 27692986

[102] Zhang HY Liao BW Xu ZS Ran Y Wang DP Yang Y. USP44 Positively Regulates Innate Immune Response to DNA Viruses Through Deubiquitinating MITA. PloS Pathog (2020) 16(1):e1008178. doi: 10.1371/journal.ppat.1008178 31968013 PMC6975528

[103] Ye L Zhang Q Liuyu T Xu Z Zhang MX Luo MH. USP49 Negatively Regulates Cellular Antiviral Responses *via* Deconjugating K63-Linked Ubiquitination of MITA. PloS Pathog (2019) 15(4):e1007680. doi: 10.1371/journal.ppat.1007680 30943264 PMC6464240

[104] Panda S Nilsson JA Gekara NO. Deubiquitinase MYSM1 Regulates Innate Immunity Through Inactivation of TRAF3 and TRAF6 Complexes. Immunity (2015) 43(4):647–59. doi: 10.1016/j.immuni.2015.09.010 26474655

[105] Tian M Liu W Zhang Q Huang Y Li W Wang W. MYSM1 Represses Innate Immunity and Autoimmunity Through Suppressing the cGAS-STING Pathway. Cell Rep (2020) 33(3):108297. doi: 10.1016/j.celrep.2020.108297 33086059

[106] Liang J Saad Y Lei T Wang J Qi D Yang Q. MCP-Induced Protein 1 Deubiquitinates TRAF Proteins and Negatively Regulates JNK and NF-kappaB Signaling. J Exp Med (2010) 207(13):2959–73. doi: 10.1084/jem.20092641 PMC3005225 21115689

[107] Chen X Zhao Q Xie Q Xing Y Chen Z. MCPIP1 Negatively Regulate Cellular Antiviral Innate Immune Responses Through DUB and Disruption of TRAF3-TBK1-IKKepsilon Complex. Biochem Biophys Res Commun (2018) 503(2):830–6. doi: 10.1016/j.bbrc.2018.06.083 PMC7092953 29920243

[108] Feng Q Miao Y Ge J Yuan Y Zuo Y Qian L. ATXN3 Positively Regulates Type I IFN Antiviral Response by Deubiquitinating and Stabilizing Hdac3. J Immunol (2018) 201(2):675–87. doi: 10.4049/jimmunol.1800285 29802126

[109] Zheng H Gupta V Patterson-Fortin J Bhattacharya S Katlinski K Wu J. A BRISC-SHMT Complex Deubiquitinates IFNAR1 and Regulates Interferon Responses. Cell Rep (2013) 5(1):180–93. doi: 10.1016/j.celrep.2013.08.025 PMC3813903 24075985

[110] Cheng Q Feng Q Xu Y Zuo Y Liu J Yuan Y. BRCC36 Functions Noncatalytically to Promote Antiviral Response by Maintaining STAT1 Protein Stability. Eur J Immunol (2020) 51(2):296–310. doi: 10.1002/eji.202048537 32673428

[111] Ren Y Zhao P Liu J Yuan Y Cheng Q Zuo Y. Deubiquitinase USP2a Sustains Interferons Antiviral Activity by Restricting Ubiquitination of Activated STAT1 in the Nucleus. PloS Pathog (2016) 12(7):e1005764. doi: 10.1371/journal.ppat.1005764 27434509 PMC4951015

[112] Qian G Zhu L Huang C Liu Y Ren Y Ding Y. Ubiquitin Specific Protease 5 Negatively Regulates the IFNs-Mediated Antiviral Activity *via* Targeting SMURF1. Int Immunopharmacol (2020) 87:106763. doi: 10.1016/j.intimp.2020.106763 32683298

[113] Yuan Y Miao Y Zeng C Liu J Chen X Qian L. Small-Molecule Inhibitors of Ubiquitin-Specific Protease 7 Enhance Type-I Interferon Antiviral Efficacy by Destabilizing SOCS1. Immunology (2020) 159(3):309–21. doi: 10.1111/imm.13147 PMC7011631 31691271

[114] Liu J Jin L Chen X Yuan Y Zuo Y Miao Y. USP12 Translocation Maintains Interferon Antiviral Efficacy by Inhibiting CBP Acetyltransferase Activity. PloS Pathog (2020) 16(1):e1008215. doi: 10.1371/journal.ppat.1008215 31899788 PMC6961928

[115] Yeh HM Yu CY Yang HC Ko SH Liao CL Lin YL. Ubiquitin-Specific Protease 13 Regulates IFN Signaling by Stabilizing STAT1. J Immunol (2013) 191(6):3328–36. doi: 10.4049/jimmunol.1300225 23940278

[116] Sarasin-Filipowicz M Wang X Yan M Duong FH Poli V Hilton DJ. Alpha Interferon Induces Long-Lasting Refractoriness of JAK-STAT Signaling in the Mouse Liver Through Induction of USP18/UBP43. Mol Cell Biol (2009) 29(17):4841–51. doi: 10.1128/MCB.00224-09 PMC2725724 19564419

[117] Malakhova OA Kim KI Luo JK Zou W Kumar KG Fuchs SY. UBP43 Is a Novel Regulator of Interferon Signaling Independent of Its ISG15 Isopeptidase Activity. EMBO J (2006) 25(11):2358–67. doi: 10.1038/sj.emboj.7601149 PMC1478183 16710296

[118] Peng Y Guo J Sun T Fu Y Zheng H Dong C. USP39 Serves as a Deubiquitinase to Stabilize STAT1 and Sustains Type I IFN-Induced Antiviral Immunity. J Immunol (2020) 205(11):3167–78. doi: 10.4049/jimmunol.1901384 33127822

[119] Qian L Zuo Y Deng W Miao Y Liu J Yuan Y. MCPIP1 is a Positive Regulator of Type I Interferons Antiviral Activity. Biochem Biophys Res Commun (2018) 498(4):891–7. doi: 10.1016/j.bbrc.2018.03.076 29545178

[120] Wang X Zhang L Zhang Y Zhao P Qian L Yuan Y. JOSD1 Negatively Regulates Type-I Interferon Antiviral Activity by Deubiquitinating and Stabilizing Socs1. Viral Immunol (2017) 30(5):342–9. doi: 10.1089/vim.2017.0015 28355105

[121] Muromoto R Nakajima M Hirashima K Hirao T Kon S Shimoda K. Jun Activation Domain-Binding Protein 1 (JAB1) Is Required for the Optimal Response to Interferons. J Biol Chem (2013) 288(43):30969–79. doi: 10.1074/jbc.M113.485847 PMC3829410 24043623

[122] Zhao P Guo T Qian L Wang X Yuan Y Cheng Q. Ubiquitin C-Terminal Hydrolase-L3 Promotes Interferon Antiviral Activity by Stabilizing Type I-Interferon Receptor. Antiviral Res (2017) 144:120–9. doi: 10.1016/j.antiviral.2017.06.002 28583475

[123] Wang Y Wang F. Post-Translational Modifications of Deubiquitinating Enzymes: Expanding the Ubiquitin Code. Front Pharmacol (2021) 12:685011. doi: 10.3389/fphar.2021.685011 34177595 PMC8224227

[124] Liu B Zhang M Chu H Zhang H Wu H Song G. The Ubiquitin E3 Ligase TRIM31 Promotes Aggregation and Activation of the Signaling Adaptor MAVS Through Lys63-Linked Polyubiquitination. Nat Immunol (2017) 18(2):214–24. doi: 10.1038/ni.3641 27992402

[125] Paz S Vilasco M Arguello M Sun Q Lacoste J Nguyen TL. Ubiquitin-Regulated Recruitment of IkappaB Kinase Epsilon to the MAVS Interferon Signaling Adapter. Mol Cell Biol (2009) 29(12):3401–12. doi: 10.1128/MCB.00880-08 PMC2698723 19380491

[126] Yoo YS Park YY Kim JH Cho H Kim SH Lee HS. The Mitochondrial Ubiquitin Ligase MARCH5 Resolves MAVS Aggregates During Antiviral Signalling. Nat Commun (2015) 6:7910. doi: 10.1038/ncomms8910 26246171 PMC4918326

[127] Song G Liu B Li Z Wu H Wang P Zhao K. E3 Ubiquitin Ligase RNF128 Promotes Innate Antiviral Immunity Through K63-Linked Ubiquitination of TBK1. Nat Immunol (2016) 17(12):1342–51. doi: 10.1038/ni.3588 27776110

[128] Nakhaei P Mesplede T Solis M Sun Q Zhao T Yang L. The E3 Ubiquitin Ligase Triad3A Negatively Regulates the RIG-I/MAVS Signaling Pathway by Targeting TRAF3 for Degradation. PloS Pathog (2009) 5(11):e1000650. doi: 10.1371/journal.ppat.1000650 19893624 PMC2766052

[129] Cui J Li Y Zhu L Liu D Songyang Z Wang HY. NLRP4 Negatively Regulates Type I Interferon Signaling by Targeting the Kinase TBK1 for Degradation *via* the Ubiquitin Ligase DTX4. Nat Immunol (2012) 13(4):387–95. doi: 10.1038/ni.2239 PMC3760161 22388039

[130] Zhang M Wang L Zhao X Zhao K Meng H Zhao W. TRAF-Interacting Protein (TRIP) Negatively Regulates IFN-Beta Production and Antiviral Response by Promoting Proteasomal Degradation of TANK-Binding Kinase 1. J Exp Med (2012) 209(10):1703–11. doi: 10.1084/jem.20120024 PMC3457734 22945920

[131] Zheng H Qian J Varghese B Baker DP Fuchs S. Ligand-Stimulated Downregulation of the Alpha Interferon Receptor: Role of Protein Kinase D2. Mol Cell Biol (2011) 31(4):710–20. doi: 10.1128/MCB.01154-10 PMC3028644 21173164

[132] Carbone CJ Zheng H Bhattacharya S Lewis JR Reiter AM Henthorn P. Protein Tyrosine Phosphatase 1B Is a Key Regulator of IFNAR1 Endocytosis and a Target for Antiviral Therapies. Proc Natl Acad Sci USA (2012) 109(47):19226–31. doi: 10.1073/pnas.1211491109 PMC3511142 23129613

[133] Tanaka T Soriano MA Grusby MJ. SLIM is a Nuclear Ubiquitin E3 Ligase That Negatively Regulates STAT Signaling. Immunity (2005) 22(6):729–36. doi: 10.1016/j.immuni.2005.04.008 15963787

[134] Yuan C Qi J Zhao X Gao C. Smurf1 Protein Negatively Regulates Interferon-Gamma Signaling Through Promoting STAT1 Protein Ubiquitination and Degradation. J Biol Chem (2012) 287(21):17006–15. doi: 10.1074/jbc.M112.341198 PMC3366787 22474288

[135] Piganis RA De Weerd NA Gould JA Schindler CW Mansell A Nicholson SE. Suppressor of Cytokine Signaling (SOCS) 1 Inhibits Type I Interferon (IFN) Signaling *via* the Interferon Alpha Receptor (IFNAR1)-Associated Tyrosine Kinase Tyk2. J Biol Chem (2011) 286(39):33811–8. doi: 10.1074/jbc.M111.270207 PMC3190811 21757742

[136] Basters A Geurink PP El Oualid F Ketscher L Casutt MS Krause E. Molecular Characterization of Ubiquitin-Specific Protease 18 Reveals Substrate Specificity for Interferon-Stimulated Gene 15. FEBS J (2014) 281(7):1918–28. doi: 10.1111/febs.12754 24533902

[137] Dauphinee SM Richer E Eva MM McIntosh F Paquet M Dangoor D. Contribution of Increased ISG15, ISGylation and Deregulated Type I IFN Signaling in Usp18 Mutant Mice During the Course of Bacterial Infections. Genes Immun (2014) 15(5):282–92. doi: 10.1038/gene.2014.17 PMC4111656 24807690

[138] Goldmann T Zeller N Raasch J Kierdorf K Frenzel K Ketscher L. USP18 Lack in Microglia Causes Destructive Interferonopathy of the Mouse Brain. EMBO J (2015) 34(12):1612–29. doi: 10.15252/embj.201490791 PMC4475397 25896511

[139] Wagner SA Beli P Weinert BT Nielsen ML Cox J Mann M. A Proteome-Wide, Quantitative Survey of *In Vivo* Ubiquitylation Sites Reveals Widespread Regulatory Roles. Mol Cell Proteomics (2011) 10(10):M111 013284. doi: 10.1074/mcp.M111.013284 PMC3205876 21890473

[140] Jiang X Chen ZJ. The Role of Ubiquitylation in Immune Defence and Pathogen Evasion. Nat Rev Immunol (2011) 12(1):35–48. doi: 10.1038/nri3111 22158412 PMC3864900

[141] Clague MJ Urbe S Komander D. Breaking the Chains: Deubiquitylating Enzyme Specificity Begets Function. Nat Rev Mol Cell Biol (2019) 20(6):338–52. doi: 10.1038/s41580-019-0099-1 30733604

[142] Kulathu Y Komander D. Atypical Ubiquitylation - the Unexplored World of Polyubiquitin Beyond Lys48 and Lys63 Linkages. Nat Rev Mol Cell Biol (2012) 13(8):508–23. doi: 10.1038/nrm3394 22820888

[143] Ebner P Versteeg GA Ikeda F. Ubiquitin Enzymes in the Regulation of Immune Responses. Crit Rev Biochem Mol Biol (2017) 52(4):425–60. doi: 10.1080/10409238.2017.1325829 PMC5490640 28524749

[144] Zong Z Zhang Z Wu L Zhang L Zhou F. The Functional Deubiquitinating Enzymes in Control of Innate Antiviral Immunity. Adv Sci (Weinh) (2021) 8(2):2002484. doi: 10.1002/advs.202002484 33511009 PMC7816709

[145] Zeng W Xu M Liu S Sun L Chen ZJ. Key Role of Ubc5 and Lysine-63 Polyubiquitination in Viral Activation of IRF3. Mol Cell (2009) 36(2):315–25. doi: 10.1016/j.molcel.2009.09.037 PMC2779157 19854139

[146] Song K Li S. The Role of Ubiquitination in NF-kappaB Signaling During Virus Infection. Viruses (2021) 13(2):1–17. doi: 10.3390/v13020145 PMC7908985 33498196

[147] Ohtake F Saeki Y Ishido S Kanno J Tanaka K. The K48-K63 Branched Ubiquitin Chain Regulates NF-kappaB Signaling. Mol Cell (2016) 64(2):251–66. doi: 10.1016/j.molcel.2016.09.014 27746020

[148] Chen J Chen ZJ. Regulation of NF-kappaB by Ubiquitination. Curr Opin Immunol (2013) 25(1):4–12. doi: 10.1016/j.coi.2012.12.005 23312890 PMC3594545

[149] Wertz IE O’Rourke KM Zhou H Eby M Aravind L Seshagiri S. De-Ubiquitination and Ubiquitin Ligase Domains of A20 Downregulate NF-kappaB Signalling. Nature (2004) 430(7000):694–9. doi: 10.1038/nature02794 15258597

[150] Chen ZJ Sun LJ. Nonproteolytic Functions of Ubiquitin in Cell Signaling. Mol Cell (2009) 33(3):275–86. doi: 10.1016/j.molcel.2009.01.014 19217402

[151] Jiang X Kinch LN Brautigam CA Chen X Du F Grishin NV. Ubiquitin-Induced Oligomerization of the RNA Sensors RIG-I and MDA5 Activates Antiviral Innate Immune Response. Immunity (2012) 36(6):959–73. doi: 10.1016/j.immuni.2012.03.022 PMC3412146 22705106

[152] van Huizen M Kikkert M. The Role of Atypical Ubiquitin Chains in the Regulation of the Antiviral Innate Immune Response. Front Cell Dev Biol (2019) 7:392. doi: 10.3389/fcell.2019.00392 32039206 PMC6987411

[153] Niu J Shi Y Xue J Miao R Huang S Wang T. USP10 Inhibits Genotoxic NF-kappaB Activation by MCPIP1-Facilitated Deubiquitination of NEMO. EMBO J (2013) 32(24):3206–19. doi: 10.1038/emboj.2013.247 PMC3981146 24270572

[154] Parvatiyar K Barber GN Harhaj EW. TAX1BP1 and A20 Inhibit Antiviral Signaling by Targeting TBK1-IKKi Kinases. J Biol Chem (2010) 285(20):14999–5009. doi: 10.1074/jbc.M110.109819 PMC2865285 20304918

[155] Shembade N Ma A Harhaj EW. Inhibition of NF-kappaB Signaling by A20 Through Disruption of Ubiquitin Enzyme Complexes. Science (2010) 327(5969):1135–9. doi: 10.1126/science.1182364 PMC3025292 20185725

[156] Tokunaga F Nishimasu H Ishitani R Goto E Noguchi T Mio K. Specific Recognition of Linear Polyubiquitin by A20 Zinc Finger 7 Is Involved in NF-kappaB Regulation. EMBO J (2012) 31(19):3856–70. doi: 10.1038/emboj.2012.241 PMC3463848 23032187

[157] Verhelst K Carpentier I Kreike M Meloni L Verstrepen L Kensche T. A20 Inhibits LUBAC-Mediated NF-kappaB Activation by Binding Linear Polyubiquitin Chains *via* its Zinc Finger 7. EMBO J (2012) 31(19):3845–55. doi: 10.1038/emboj.2012.240 PMC3463847 23032186

[158] Harrigan JA Jacq X Martin NM Jackson SP. Deubiquitylating Enzymes and Drug Discovery: Emerging Opportunities. Nat Rev Drug Discovery (2018) 17(1):57–78. doi: 10.1038/nrd.2017.152 28959952 PMC7097658

[159] Schauer NJ Magin RS Liu X Doherty LM Buhrlage SJ. Advances in Discovering Deubiquitinating Enzyme (DUB) Inhibitors. J Med Chem (2020) 63(6):2731–50. doi: 10.1021/acs.jmedchem.9b01138 31682427

[160] Tomala MD Magiera-Mularz K Kubica K Krzanik S Zieba B Musielak B. Identification of Small-Molecule Inhibitors of USP2a. Eur J Med Chem (2018) 150:261–7. doi: 10.1016/j.ejmech.2018.03.009 29529503

[161] Lopez-Castejon G Luheshi NM Compan V High S Whitehead RC Flitsch S. Deubiquitinases Regulate the Activity of Caspase-1 and Interleukin-1beta Secretion *via* Assembly of the Inflammasome. J Biol Chem (2013) 288(4):2721–33. doi: 10.1074/jbc.M112.422238 PMC3554938 23209292

[162] Wu HQ Baker D Ovaa H. Small Molecules That Target the Ubiquitin System. Biochem Soc Trans (2020) 48(2):479–97. doi: 10.1042/BST20190535 PMC7200645 32196552

[163] Love KR Catic A Schlieker C Ploegh HL. Mechanisms, Biology and Inhibitors of Deubiquitinating Enzymes. Nat Chem Biol (2007) 3(11):697–705. doi: 10.1038/nchembio.2007.43 17948018

[164] Catrysse L Vereecke L Beyaert R van Loo G. A20 in Inflammation and Autoimmunity. Trends Immunol (2014) 35(1):22–31. doi: 10.1016/j.it.2013.10.005 24246475

[165] Ruan J Schluter D Wang X. Deubiquitinating Enzymes (DUBs): DoUBle-Edged Swords in CNS Autoimmunity. J Neuroinflamm (2020) 17(1):102. doi: 10.1186/s12974-020-01783-8 PMC7132956 32248814

[166] Meuwissen ME Schot R Buta S Oudesluijs G Tinschert S Speer SD. Human USP18 Deficiency Underlies Type 1 Interferonopathy Leading to Severe Pseudo-TORCH Syndrome. J Exp Med (2016) 213(7):1163–74. doi: 10.1084/jem.20151529 PMC4925017 27325888

[167] Alsohime F Martin-Fernandez M Temsah MH Alabdulhafid M Le Voyer T Alghamdi M. JAK Inhibitor Therapy in a Child With Inherited USP18 Deficiency. New Engl J Med (2020) 382(3):256–65. doi: 10.1056/NEJMoa1905633 PMC7155173 31940699

[168] Gruber C Martin-Fernandez M Ailal F Qiu X Taft J Altman J. Homozygous STAT2 Gain-of-Function Mutation by Loss of USP18 Activity in a Patient With Type I Interferonopathy. J Exp Med (2020) 217(5):1–9. doi: 10.1084/jem.20192319 PMC7201920 32092142

[169] Kemp M. Recent Advances in the Discovery of Deubiquitinating Enzyme Inhibitors. Prog Med Chem (2016) 55:149–92. doi: 10.1016/bs.pmch.2015.10.002 PMC7112275 26852935

[170] Altun M Kramer HB Willems LI McDermott JL Leach CA Goldenberg SJ. Activity-Based Chemical Proteomics Accelerates Inhibitor Development for Deubiquitylating Enzymes. Chem Biol (2011) 18(11):1401–12. doi: 10.1016/j.chembiol.2011.08.018 22118674

[171] Hoffmann HH Schneider WM Rice CM. Interferons and Viruses: An Evolutionary Arms Race of Molecular Interactions. Trends Immunol (2015) 36(3):124–38. doi: 10.1016/j.it.2015.01.004 PMC4384471 25704559

[172] Bailey-Elkin BA Knaap RCM Kikkert M Mark BL. Structure and Function of Viral Deubiquitinating Enzymes. J Mol Biol (2017) 429(22):3441–70. doi: 10.1016/j.jmb.2017.06.010 PMC7094624 28625850

